# FOXM1 modulates 5-FU resistance in colorectal cancer through regulating TYMS expression

**DOI:** 10.1038/s41598-018-38017-0

**Published:** 2019-02-06

**Authors:** Vidhya Varghese, Luca Magnani, Narumi Harada-Shoji, Francesco Mauri, Richard M. Szydlo, Shang Yao, Eric W.-F. Lam, Laura M. Kenny

**Affiliations:** 1 Department of Surgery and Cancer, Imperial College London, London, USA; 2 Imperial College Healthcare NHS Trust, Imperial College London, London, USA; 3 Department of Haematology, Imperial College London, London, USA

## Abstract

Resistance to 5-Fluoruracil (5-FU) has been linked to elevated expression of the main target, thymidylate synthase (TYMS), which catalyses the *de novo* pathway for production of deoxythymidine monophosphate. The potent oncogenic forkhead box transcription factor, FOXM1 is is regulated by E2F1 which also controls TYMS. This study reveals a significant role of FOXM1 in 5-FU resistance. Overexpression and knock-down studies of FOXM1 in colon cancer cells suggest the importance of FOXM1 in TYMS regulation. ChIP and global ChIP-seq data also confirms that FOXM1 can also potentially regulate other 5-FU targets, such as TYMS, thymidine kinase 1 (TK-1) and thymidine phosphorylase (TYMP). In human colorectal cancer tissue specimens, a strong correlation of FOXM1 and TYMS staining was observed. Elevated FOXM1 and TYMS expression was also observed in acquired 5-FU resistant colon cancer cells (HCT116 5-FU Res). A synergistic effect was observed following treatment of CRC cells with an inhibitor of FOXM1, thiostrepton, in combination with 5-FU. The combination treatment decreased colony formation and migration, and induced cell cycle arrest, DNA damage, and apoptosis in CRC cell lines. In summary, this research demonstrated that FOXM1 plays a pivotal role in 5-FU resistance at least partially through the regulation of TYMS.

## Introduction

Colorectal cancer (CRC) is a leading cause of cancer mortality, andcurrent strategies for treating this condition need to be improved^1,2^. Fluoropyrimidine, 5-Flourouracil (5-FU), is the most commonly used drug in the clinical treatment of CRC today, and forms the backbone of all first-line therapy both for adjuvant and metastatic treatments^3,4^. Resistance to treatment is common, especially in the metastatic setting, and understanding the mechanisms which regulate the targets of 5-FU could help identifying novel treatment strategies to improve patient outcomes.

The main target of 5-FU is the thymidylate synthase enzyme (TYMS) (EC 2.1.1.4.5), which catalyzes the formation of deoxythymidine-5′-monophosphate (dTMP) from 2′-deoxyuridine monophosphate using 5′10-methylene tetrahydrofolate as a cofactor via the de novo pathway; dTMP is an essential precursor for DNA synthesis^5,6^. Overexpression of TYMS is linked to resistance to TYMS targeted drugs such as 5-FU in both breast and colorectal cancer^7^. Similarly, low levels of TYMS in CRC predicted a good response rate to 5-FU and a significantly longer survival in patients with advanced colorectal carcinoma^8^. Consistently, higher TYMS expression is found in resistant colon cancer cells compared to sensitive colon cancer cell lines^9,10^. Patients with tumours expressing high levels of TYMS have a poorer OS (overall survival) compared with those with tumours expressing low levels of TYMS^9,10^. Furthermore tumour samples with high TYMS levels are more likely to be resistant to 5-FU^11^. Conversely, increased levels of TYMS expression in clinical CRC specimens have been shown to predict poorresponse to 5-FU^12–14^. Although some conflicting results have been observed in clinical trials, they are thought to be due to a lack of standardised methodologies^15^. Another molecule involved in 5-FU response is p53. Studies have shown that cells with wild-type p53 are more sensitive to 5-FU compared to p53 mutant cells which undergo significantly lower levels of apoptosis in response to 5-FU^16^. It is well known that the E2F1 transcription factor regulates the cell cycle and induces DNA synthesis, by controlling G1-S regulatory genes, including TYMS and the forkhead box transcription factor, FOXM1^17–19^.

Emerging evidence suggests that elevated FOXM1 levels promote cancer progression and are associated with a variety of aggressive and chemotherapy resistant human cancers^20^. In colorectal cancer, FOXM1 has been shown to be involved in carcinogenesis using a Rosa26-FOXM1 transgenic mouse model. These FOXM1-transgenic mice display increased growth and higher numbers of tumours compared to wild-type controls. Conversely, FOXM1 depletion is associated with reduced CRC carcinogenesis and growth after exposure to carcinogens^21^. Elevated expression of FOXM1 has been found in human CRC compared to matched normal tissues^22^. However, little is known about the role of FOXM1 in colorectal cancer, specifically with respect to 5-FU resistance.

Here, for the first time, we investigated the role of FOXM1 in relation to 5-FU resistance in colorectal cancer cells *in vitro* using p53 wild-type and mutant CRC cells as well as 5-FU sensitive and resistant CRC cells.

## Results

### TYMS expression and its direct association with FOXM1 in patients with colon cancer

**Figure 1:**
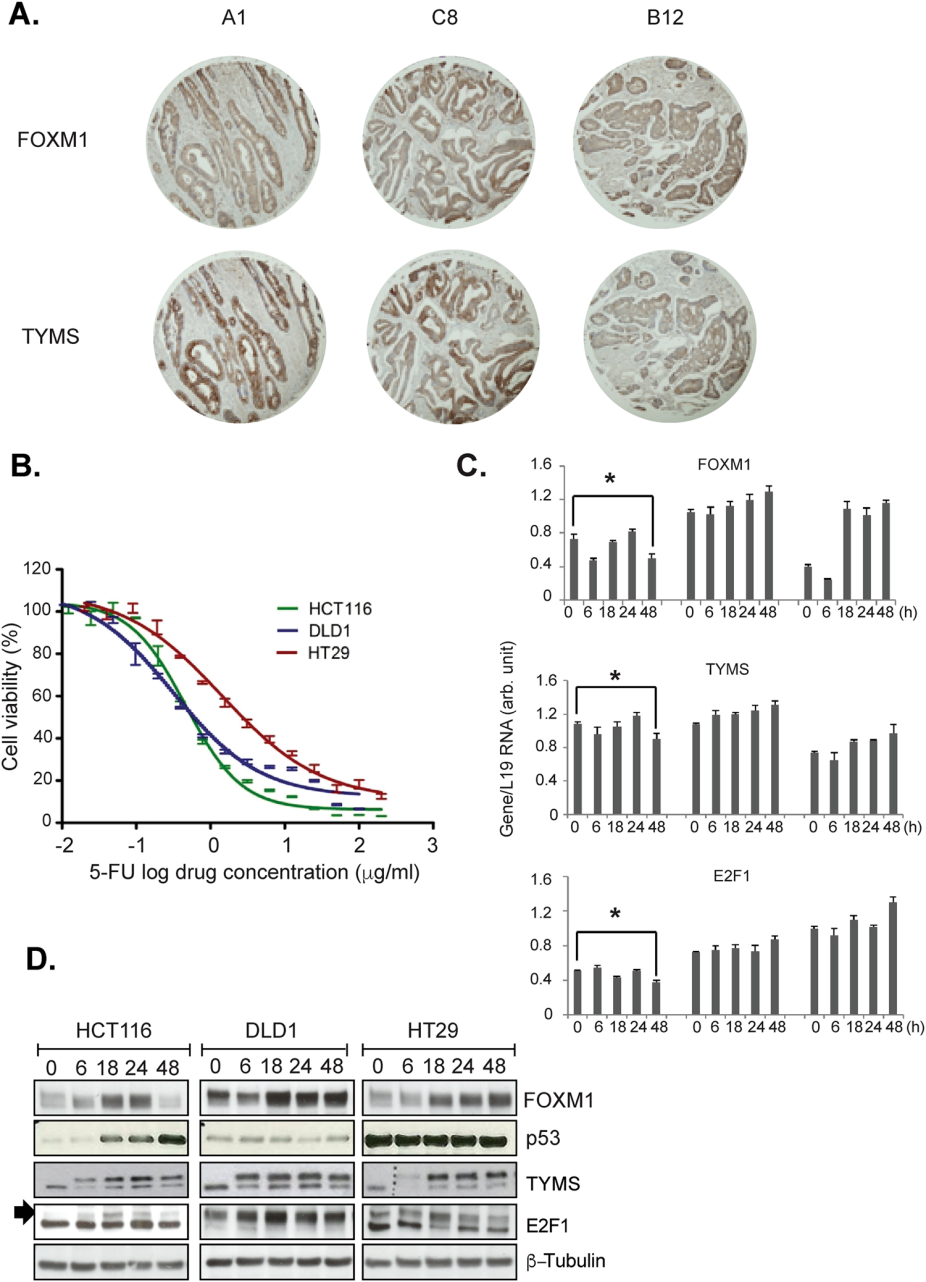
Correlations between FOXM1 and TYMS in CRC. (**A**) Representative photographs of FOXM1 and TYMS, staining in human CRC tissue showing close similarity in protein expression patterns. Cytoplasmic FOXM1 expression levels were directly correlated with the TYMS expression in the TMA (p = 0.008, r = 0.314). The images above were taken from the A1, C8 and B12 cores: (**B**) SRB assay shows the average cell viability of colon cancer cells. HCT116, DLD1, and HT29 colon cancer cells were treated with 5-FU, ranging from 0 to 100 µg/ml for 72 h. IC50 values were: HCT116 0.6 ± 0.23, DLD1 0.7 ± 0.22, and HT29 3.09 ± 0.39 µg/ml. Results shown are mean and SD of three independent experiments, each with three replicates. IC50 values were determined by fitting a sigmoidal dose-response curve to the data using Graph Pad Prism. HCT116, DLD1, and HT29 cells were seeded at a 60–70% confluence, the cells were treated with 0.5 µg/ml 5-FU for 0, 6, 18, 24, and 48 h. Cells were trypsinised and harvested at the indicated time points for (**C**) RT-PCR and (**D**) western blot. Gene expression was quantified using a standard curve and normalised to the housekeeping gene L19. This HCT116 FOXM1, TYMS and E2F1 mRNA levels at 48 h. Error Bars represent standard deviation. Statistical significance was determined by student’s T-test (*p value < 0.05 0 h versus 48 h in HCT116 cells). The mRNA patterns were reflected in protein expression as shown in Western blots.

To study the expression and correlation of FOXM1 and TYMS in colon cancer, immunohistochemistry was performed in a commercial colorectal tumour tissue microarray of 110 colon cancer samples (Fig. 1A). In the array, we observed FOXM1 positive staining in both the cytoplasm and nuclei of the majority of cancer cells (>90%), indicating that FOXM1 is commonly overexpressed in human colon cancer. We further evaluated TYMS expression in the same cohort and observed strong TYMS positive staining in the cytoplasm of CRC cells. Furthermore, there was a significant correlation between FOXM1 and TYMS (Spearman r = 0.314, p = 0.008). These results provide the clinical evidence to support the positive relationship between FOXM1 and TYMS expression.

### FOXM1, TYMS, and E2F1 genes are associated with 5-FU response in colon cancer cells

The cytotoxic effect of 5-FU was examined in HCT116 (p53 wild-type), DLD1 (TP53 allele heterozygous mutant) and HT29 (TP53 allele homozygous mutant)^22^ cell lines using SRB assays (Fig. 1B). Treatment reduced cell viability in a time- (72 hour) and concentration-dependent (0–100 µg/ml) manner. HT29 cells were 5-fold more resistant than HCT116 or DLD1 cells (IC50 mean ± SD = 3.1 ± 0.4 µg/ml, 0.6 ± 0.2 µg/ml, 0.7 ± 0.2 µg/ml, respectively). According to a study by Boxtel RV *et al*.^23^, homozygous mutant rats that completely lack TP53 showed a decrease in survival as a result of spontaneous tumour development compared to heterozygous rats^23^. This could explain our result in which HT29 cells showed higher resistance to 5-FU than DLD1 cells, even though both harbour mutant p53. We carried out western blots to screen and detect the expression of different proteins, which could have a potential role in 5-FU resistance. Expression of FOXM1 protein and mRNA was found to be induced initially at 18–24 hours then decreased at 48 hours in 5-FU treated HCT116 cells and importantly, these kinetics were mirrored by E2F1 and TYMS protein and mRNA (Fig. 1C,D). In DLD1 and HT29 cells with mutant p53, the up-regulation of E2F1, FOXM1 and TYMS persisted until 48 h. Although p53 protein is expressed in DLD1 and HT29 cells, it is not fully functional since p53 is mutated in these cell lines are^24^. Notably, it is evident that in the DLD1 cells, the p53 levels increased at 18 h following treatment with 5-FU, consistent with the kinetics of p53 induction observed in the HCT116 and HT29 cells (Fig. 1D). This induction of WT p53 at 18 h is likely to be masked by the mutant p53 (encoded by the other mutant p53 allele) already expressed at high levels in the untreated DLD1 cells. Our results are consistent with previous studies showing that DNA damage will result in an initial induction of FOXM1 followed by an eventual down-regulation; however, in the absence of functional p53, persistent up-regulation of FOXM1 is observed during DNA damage^19^.

In agreement, in the 5-FU treated MCF7 (p53 wt) and Doxorubicin-resistant MCF7 (p53 inactive) (Supplementary Fig. S1), FOXM1 protein was found to be induced and then decreased in later time points. Downregulation of E2F1 and TYMS protein was also observed in the wild-type MCF7 cells. Similar to HCT116, p53 is augmented in MCF7 wt cells but no changes were observed in the Doxorubicin-resistant MCF7 cells. This is consistent with previous studies that demonstrated genotoxic drugs cause the activation of DNA damage genes and stabilisation of p53. Stabilisation of p53 has been shown to decrease FOXM1 expression in the p53 wt MCF7 cells^25^.

Treatment with 5-FU has been shown to induce TYMS expression^26^, This induction is due to inhibition of the auto-regulatory mechanism of TYMS by FdUMP. When stably bound by FdUMP, TYMS can no longer bind its own mRNA to suppress translation, and therefore causes an increase in protein expression. The double band observed after 5-FU treatment in TYMS (ternary complex-upper band and the free- TYMS lower band) is due to the active metabolite of 5-FU, FdUMP, forming a ternary complex with TYMS and its reduced-folate cofactor^27^.

### FOXM1 increases chemoresistance to 5-FU via TYMS

**Figure 2:**
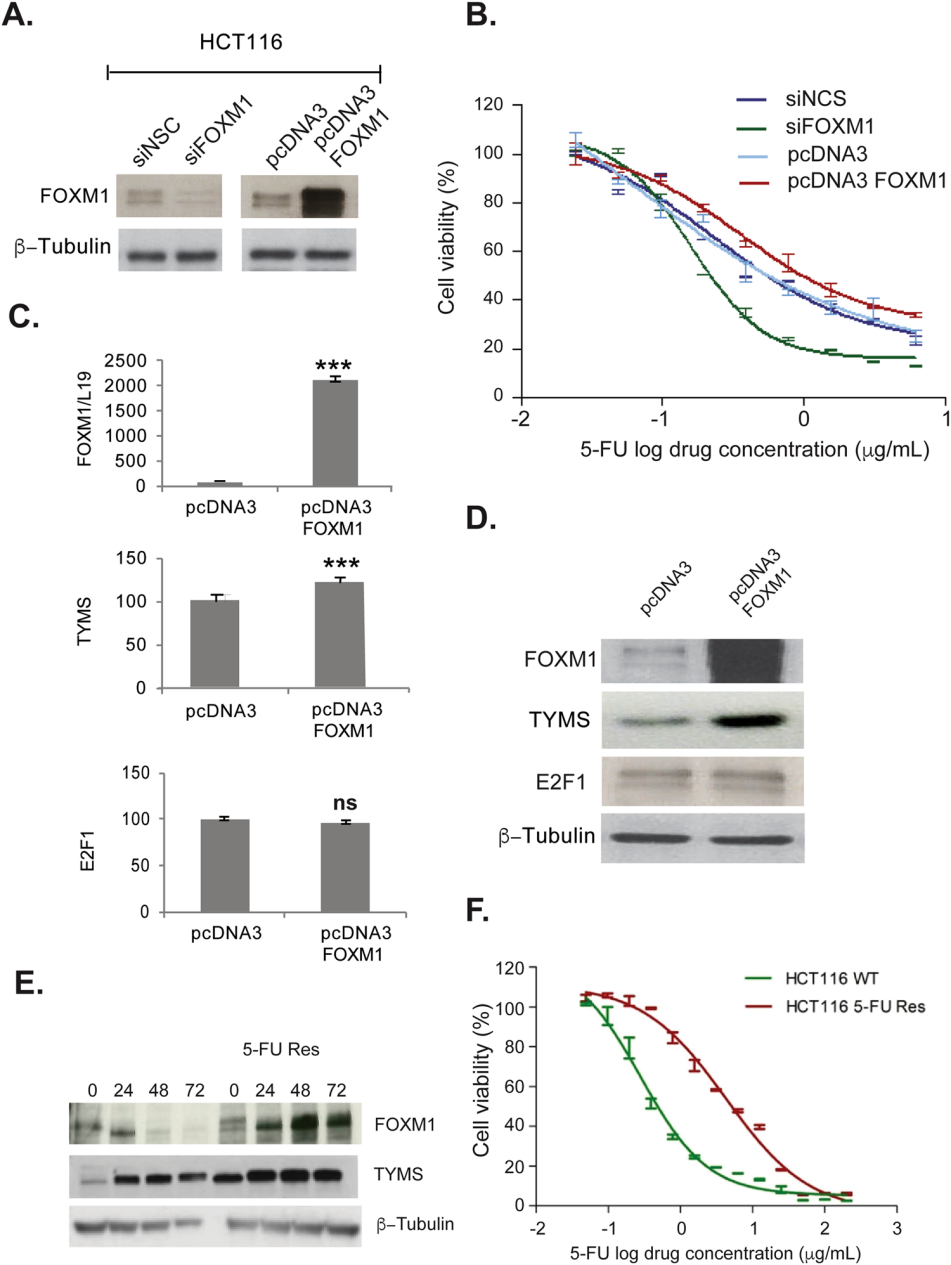
Overexpression of FOXM1 increases chemo-resistance to 5-FU. (**A**) HCT116 cells were transiently transfected with SiFOXM1 or FOXM1 pcDNA3 plasmid, after 48 h transfection cells were harvested and probed for FOXM1 by Western blotting. (**B**) HCT116 cells were transiently transfected with SiFOXM1 and FOXM1 pcDNA3 plasmid. After 48 h transfection cells were harvested and seeded in 96 well plate and treated with 5-FU for 72 h. Overexpressed FOXM1 increases chemoresistance in HCT116 cells measured using SRB assays, whereas SiFOXM1 HCT116 cells were more sensitive to treatment. Over-expression of FOXM1 is associated with increased TYMS mRNA (**C**) levels and protein expression (**D**) but did not affect E2F1. (**E**) HCT116 cells with acquired resistance to 5-FU (HCT116 5-FU Res) have higher basal expression of FOXM1 and TYMS compared to wild-type HCT116 cells, TYMS and FOXM1 remain elevated in the resistant cell line following treatment with 0.5 µg/ml of 5-FU. F) The IC50s show a 10 fold difference in sensitivity to 5-FU between the two cell lines (mean IC50, 0.54 μg/ml ± SD 0.03, n = 2) and HCT116 5-FU Res cells (mean IC50, 5.33 μg/ml ± SD 0.30, n = 3).

Next, HCT116 cells were transiently transfected with siFOXM1 or FOXM1 pcDNA3 plasmid and treated with 5-FU in comparison to HCT116 control cells. Transient expression of pCDNA3 FOXM1 was found to be associated with 5-FU resistance compared to controls (p = 0.003). FOXM1 knock-down cells also became significantly more sensitive towards 5-FU compared to controls (p = 0.0001) (Fig. 2A,B). Overexpression of FOXM1 (Fig. 2C,D) significantly increased both protein and mRNA expression of TYMS, but no changes were observed in E2F1 protein or mRNA level.

We then compared FOXM1 expression in HCT116 wt cells with HCT116 cells acquired 5-FU resistance. 5-FU resistant HCT116 cells were 10 fold more resistant to 5-FU than HCT116 wt cells (Fig. 2F). Interestingly, basal expression of FOXM1 and TYMS were found to be high in acquired 5-FU resistant cells, and persistent up-regulation was observed following 5-FU treatment compared to wild-type. These results strongly suggest that FOXM1 has a role in 5-FU resistance in colorectal cancer and that this could be mediated via TYMS (Fig. 2E).

As TYMS is known as a critical enzyme for DNA replication and cell growth, we decided to knockdown TYMS in HT29 and DLD1 cells which express high levels of FOXM1 (Supplementary Fig. S3A,B). Cell viability assay showed that knockdown of TYMS did not affect the sensitivity to 5-FU in these CRC cells expressing high levels of FOXM1. Next, we co-transfected pcDNA3 FOXM1 and SiTYMS in HCT116 wt cells (Supplementary Fig. S3) and the result showed that overexpression of FOXM1 increased resistance to 5-FU in the presence of TYMS knockdown. Indeed, western blotting results showed that overexpression of FOXM1 and silencing of TYMS using siRNA resulted in an increase in FOXM1 expression and a decrease in TYMS levels in HTC116 cells. The proliferation assays showed that overexpression of FOXM1 increased 5-FU resistance despite a downregulation in TYMS expression induced by siTYMS. This is consistent with the results from TYMS depletion in the FOXM1 overexpressing DLD1 and HT29 cells, indicating that TYMS is not the sole target of FOXM1 in mediating 5-FU resistance.

**Figure 3:**
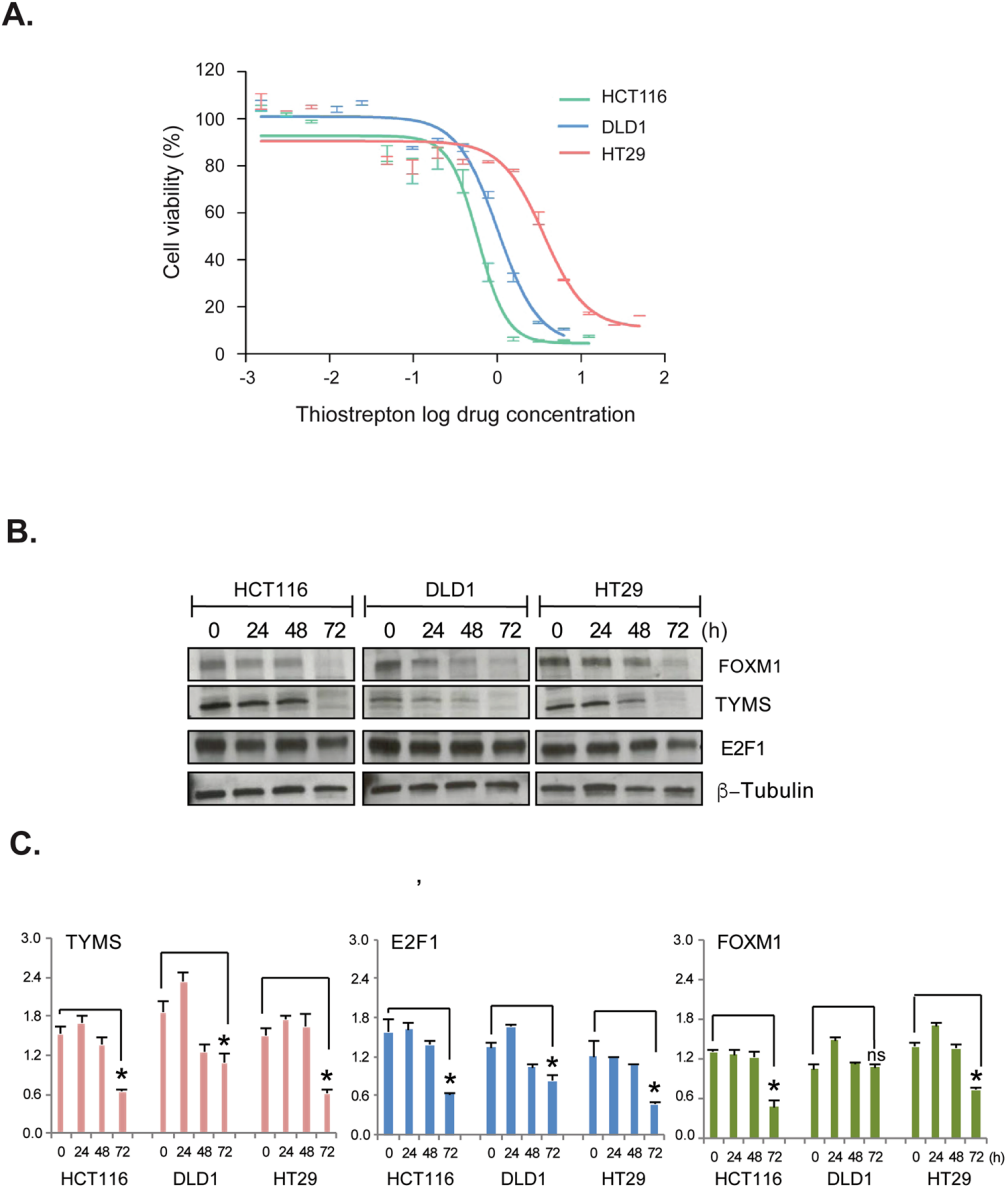
FOXM1 regulates TYMS in colon cancer. (**A**) Cells were treated with the FOXM1 inhibitor thiostrepton to detect cell viability. HCT116 IC50-0.65 ± 0.37 μM, DLD1 IC50-1.43 ± 0.02 μM, HT29 IC50-2.1 ± 0.98 μM. (**B**,**C**) HCT116, DLD1, and HT29 colon cancer cells were treated with 2 µM of thiostrepton for 0, 24, 48, and 72 h; cells were trypsinised and harvested at the time points indicated for western blot analysis and RT-PCR. (**A**) Protein lysates were prepared and expression levels were analysed by western blotting using antibodies for FOXM1, E2F1, TYMS and TK-1. β-tubulin was used as a loading control. mRNA expression indicates a significant decrease in FOXM1, TYMS, E2F1 and TK-1 levels (**B**–**E**) at 72 h. Error Bars represent standard deviation. Statistical significance was determined by student’s T-test. (*p value < 0.05 versus control).

Previous studies have shown that thiostrepton directly interacts with FOXM1 and inhibits its binding to genomic targets, such as MYC, CDC25B, CCNB1, XBP1, CCNB1 and PLK1^28,29^. We therefore decided to study the expression of FOXM1, TYMS, and E2F1 in colon cancer cell lines, following treatment with 2 µM of thiostrepton for 24, 48 and 72 hours. (Fig. 3A–C). After thiostrepton treatment, significant reductions in FOXM1 and TYMS protein and mRNA were observed. The mRNA expression patterns of TYMS were similar to FOXM1. Slight decreases were observed in E2F1 protein expression at 72 h in the HCT116 and HT29 cell lines, and significant downregulation of E2F1 mRNA was observed in the HCT116 and HT29 cells but no drastic changes were observed for DLD1, and the reason for this is unclear.

Furthermore, The IC50 value for HCT116 5-FU Res cells to thiostrepton (0.8 µM) was similar to that of HCT116 wt (Supplementary Fig. S2). This result clearly indicates that FOXM1 is essential for 5-FU resistance and that inhibiting FOXM1 alone can be cytotoxic even in the 5-FU resistant cells.

### FOXM1 binds to the Thymidylate Synthase (TYMS) promoter region

ChIP (Chromatin immunoprecipitation) assay was performed to examine if FOXM1 can directly bind to the *TYMS* promoter region in HCT116, DLD1 and HCT116 5-FU Res cell lines.

**Figure 4:**
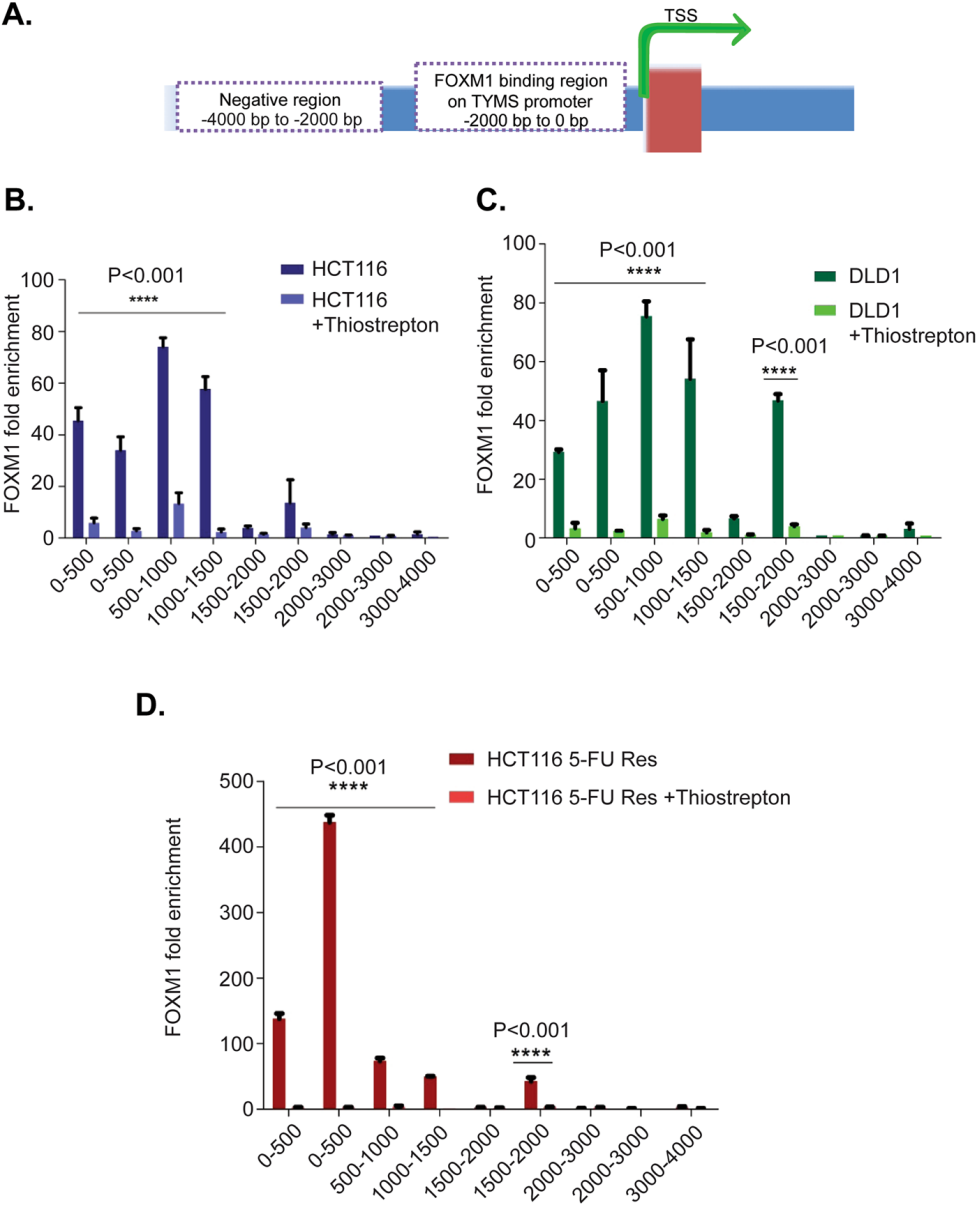
FOXM1 binding site on Thymidylate Synthase (TYMS) promoter region. (**A**) Schematic representation of FOXM1 binding sites in 0 to 2000 bp upstream of TYMS transcription start site (TSS). ChIP for FOXM1 was done followed by RT PCR to analyses the binding in the TYMS promoter region in HCT116 (**B**), DLD1 (**C**), HCT116 5-FU res (**D**) cell lines. Enrichment of FOXM1 at the TYMS promoter (0–2000 bp upstream) and no enrichment observed in the control (2000–4000 bp). Thiostrepton treatment significantly down-regulates FOXM1 binding on TYMS promoter. These experiments were done in triplicate. Error Bars represent standard deviation. Statistical significance was determined by 2 way annova using graph pad prism. (P value < 0.001).

Six sets of TYMS primers from 0 to 2000 base pair (bp) upstream and 3 control primers from 2000 bp to 4000 bp upstream of TYMS transcription start site were designed. A schematic representation of the primers and the FOXM1 binding sites on the TYMS transcription start site (TSS) is shown in Fig. 4A.

Enrichment of FOXM1 binding was observed in 0–2000 bp upstream of the TYMS promoter region, suggesting that FOXM1 can directly regulate *TYMS* expression. No enrichment was observed in the controls (2000–4000 bp). (Fig. 4B,C and D). Interestingly, expression of FOXM1 was significantly higher in the acquired 5-FU resistant HCT116 cells compared to the HCT116 wt and DLD1 cells. This experiment suggests direct regulation of TYMS by FOXM1, and that FOXM1 is an important mediator of 5-FU drug resistance through TYMS.

Previous studies have showed that thiostrepton can inhibit FOXM1 DNA binding by interacting with FOXM1 directly^30^. Consistent with this idea, HCT116, DLD1, and HCT116 5-FU Res cell lines were treated with 2 µM of thiostrepton for 24 hours. Interestingly, the results showed a significant (P < 0.001) disruption in the occupancy of the *TYMS* promoter region by FOXM1 in all 3 cell lines. Specifically, decreases in FOXM1 binding of 80% in HCT116, 85% in DLD1, and 90% in HCT116 5-FU Res (Fig. 4B–D respectively) were observed in the *TYMS* promoter region.

### Genome-wide distribution of FOXM1 binding in HCT116 cells

**Figure 5:**
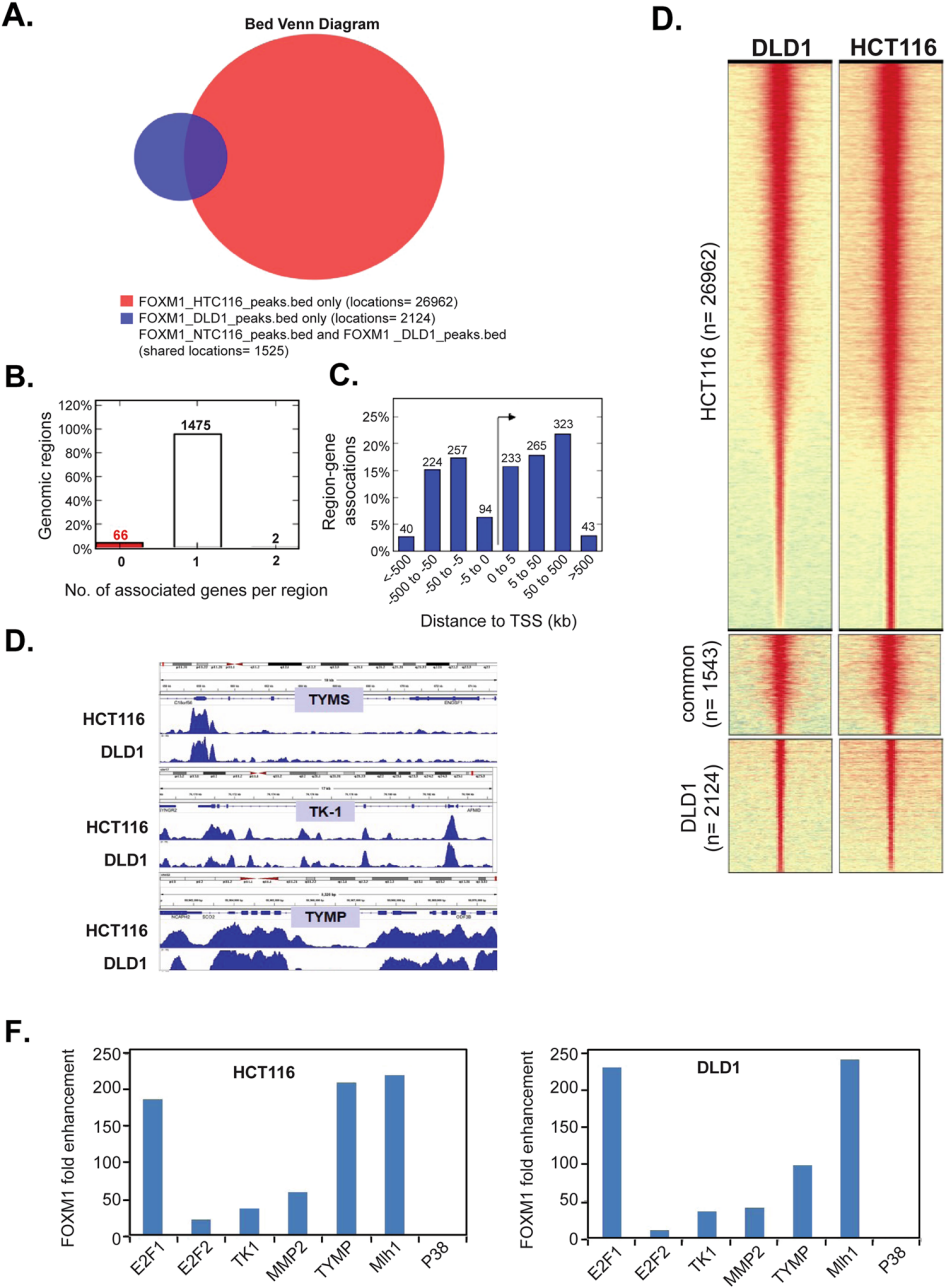
Genome wide distribution of FOXM1 binding in HCT116 cells. (**A**) Venn diagram representing the shared FOXM1 peaks between HCT116 and DLD1 cells. (**B**) Graphs displaying statistics about the association of input genomic regions to the TSS of all the genes putatively regulated by the genomic regions. The ‘Number of associated genes per region’ graph shows how many genes each genomic region is assigned as putatively regulating based on the association rule used. (**C**) The distance to TSS graphs show the distance between input regions and their putatively regulated genes. The distances are divided into four separate bins: one from 0 to 5 kb, another from 5 kb to 50 kb, a third from 50 kb to 500 kb, and a final bin of all associations over 500 Kb. (**D**) Heat map showing FOXM1 binding events in HCT116 (right) and DLD1 (left) and HCT116 DLD1 shared reads. Heatmap was generated using ChAsE (Chromatin Analysis and Exploration) platform. Settings (peak extensions 5 kb upstream and 5 kb downstream of the peak summit and bin size 50 (bp). (**E**) The Integrative Genomics Viewer (IGV) browser was used to visualise the FOXM1 binding in down-stream targets. The input track has been subtracted from the data shown above. Blue peaks represent FOXM1 enrichment in 5-FU targets: TYMS, TYMP, and TK-1. (**F**) To confirm the IGV ChIP-seq data of FOXM1 binding, RT-PCR was carried out on HCT116 (**A**) and DLD1 (**B**) cells. The result shows FOXM1 binding on TK-1, TYMP, E2F1, E2F2, MMP2, and MLH1. P38, which is not a FOXM1 regulator, does not show any FOXM1 enrichment.

To gain further insights into FOXM1 binding to other possible targets, we performed ChIP-seq analysis of FOXM1 binding in the HCT116 and DLD1 cells. Single-end 50 bp long reads were obtained from a HiSeq2000 instrument (Illumina). MACS peak-calling algorithm was used to call significantly enriched peaks using default settings (P < 10^−5^). In order to visualize the raw profiles on the UCSC Genome Browser, wiggle files were generated with MACS v1.4 and converted to bigWig. Overall number of peaks identified in HCT116 input was 1848872 and HCT116 chromatin 7636560. Overall number of peaks identified in DLD1 input was 8640633 and DLD1 chromatin 44773456. Total number of shared FOXM1 reads between HCT116 and DLD1 was determined using web-based CEAS software of the Cistrome/Galaxy platform. The venn-diagram shows intersect of FOXM1 peaks in HCT116 and DLD1 (Fig. 5A). The number of reads, observed in HCT116 was 26962, but in DLD1 few reads were observed, 2124. Total number of shared reads observed between HCT116 and DLD1 was 1525.

The distribution of FOXM1 ChIP-seq regions in HCT116 (bottom) compared to the total genomic DNA distribution (top) is shown (Fig. 5B). The bottom panel shows a detailed representation of FOXM1 binding 3 kb upstream from the promoter region (Fig. 5C). 12.4% of binding was observed in 3 kb region, and 4% binding was observed in 5′UTR, where most of the TSS are present. 37% of binding was observed in introns,where enhancers are usually present. The FOXM1 ChIP-seq showed 12.6% FOXM1 binding in 0 to 3 kb upstream of the promoter region compared to the total genome (2.5%). The distance to TSS graphs show the number of putatively FOXM1 regulated genes, with distances divided into four separate groups, one from 0 to 5 kb, another from 5 kb to 50 kb, a third from 50 kb to 500 kb, and the final is associations over 500 kb (Supplementary Fig. S4). A heat map is shown (Fig. 5D), revealing FOXM1 binding events in HCT116 and DLD1 cells. Three main motifs were identified using MEME software (Supplementary Section Fig. 7), and > 60%, matches to these motifs were found in or were located very close to the FOXM1 binding peak. A number of genes associated with this motifs are CPEB1, FOXJ3, FOXO1, HNF6, FOXL1, FUP1, and BRAC. Next, the Integrative Genomics Viewer (IGV) was used to visualise the binding of FOXM1 to other potential targets of 5-FU (Fig. 5E and Supplementary Fig. 5). Blue peaks represent FOXM1 enrichment in 5-FU targets: TYMS, TYMP, and TK-1; E2F transcription activators: E2F1, E2F2, and E2F3; Cell cycle regulatory genes: Cyclins B1, Cyclins D1, CDKN1A (p21^Cip^), CDKN1C (p57); Mismatch repair gene: MLH1; DNA damage repair gene: RAD51, RAD54, XRCC1, BRCA2 and matrix metalloproteinase(involved in progression): MMP9, MMP2 in HCT116 and DLD1 cells. Input track is not included because input was subtracted from the data before running on IGV. To confirm the IGV ChIP-seq data, expression of these genes in HCT116 and DLD1 were validated by RT-PCR analysis (Fig. 5F). The results confirmed significant enrichment in 5-FU targets such as TK-1 and TYMP, and enrichment was also observed in E2F1, E2F2, MMP2, and MLH1 (mismatch repair gene).

### Combination of thiostrepton and 5-FU increases DNA damage in colon cancer cells

**Figure 6:**
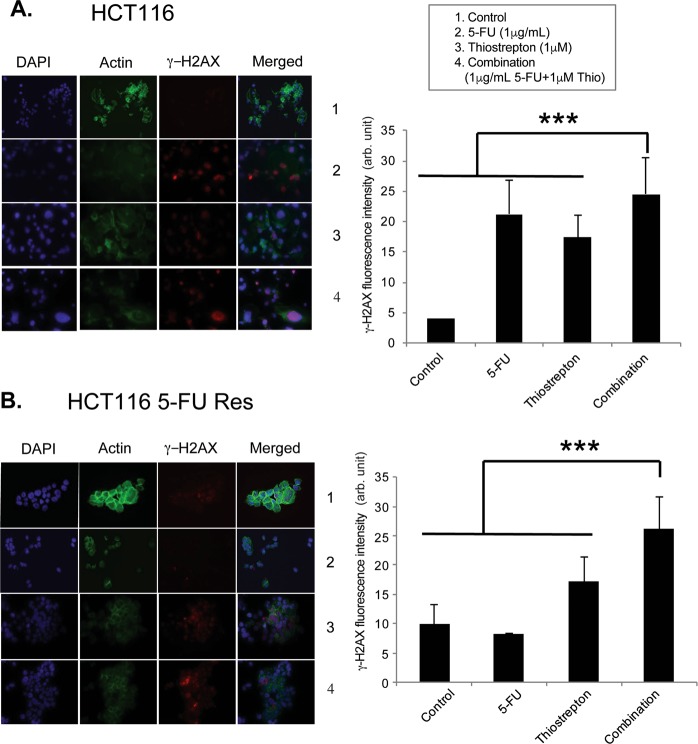
Combination of thiostrepton and 5-FU increases DNA damage in colon cancer cells. Combination of thiostrepton and 5-FU increases DNA damage in (**A**) HCT116 wt and (**B**) HCT116 5-FU res cell lines. Both cells were treated with 1 µg/ml of 5-FU, 1 µM of thiostrepton and combination for 24 h and stained with DAPI, tubulin and γH2AX antibodies. Combination of thiostrepton and 5-FU increases DNA damage in both cells. The combination of 5-FU and thiostrepton significantly increases γH2AX staining in HCT116 cells compared to control and either drugs alone ***p < 0.01. The combination of 5-FU and thiostrepton significantly increases γH2AX staining in HCT116 5-FU Res cells compared to control and either drugs alone ***p < 0.001. Images were visualized by microscopy. Images: original magnification X 40. Images were quantified using ImageJ software.

Next, we examined if the combination of 5-FU and thiostrepton treatment increases DNA damage. HCT116 wt, and HCT116 5-FU Res colon cancer cell lines were treated with 1 µg/ml 5-FU and 1 µM thiostrepton alone or in combination for 24 hours and immunostained for ɣH2AX, a known marker to detect the presence of DNA damage induced by the treatments. P53 wt HCT116 cells showed higher levels of DNA damage after the treatment with 5-FU alone compared to thiostrepton and combination treatment significantly increased DNA damage in HCT116 wt (Fig. 6A). Treatment with 5-FU alone in HCT116 5-FU Res cells resulted in no increases in DNA damage, while combination treatment led to significant higher levels of γH2AX staining in HCT116 5-FU Res cells (Fig. 6B). Quantification of γH2AX foci showed that combination treatment induced the accumulation of significantly more foci observed in both cell lines comparison to controls. This might be due to thiostrepton binding to FOXM1 to inhibit transcription of its target DNA damage repair genes, which in turns leads to the accumulation of unrepaired damaged DNA and apoptosis in response to 5-FU.

### Combination treatment inhibits colony formation and migration and enhances apoptosis in colon cancer

**Figure 7:**
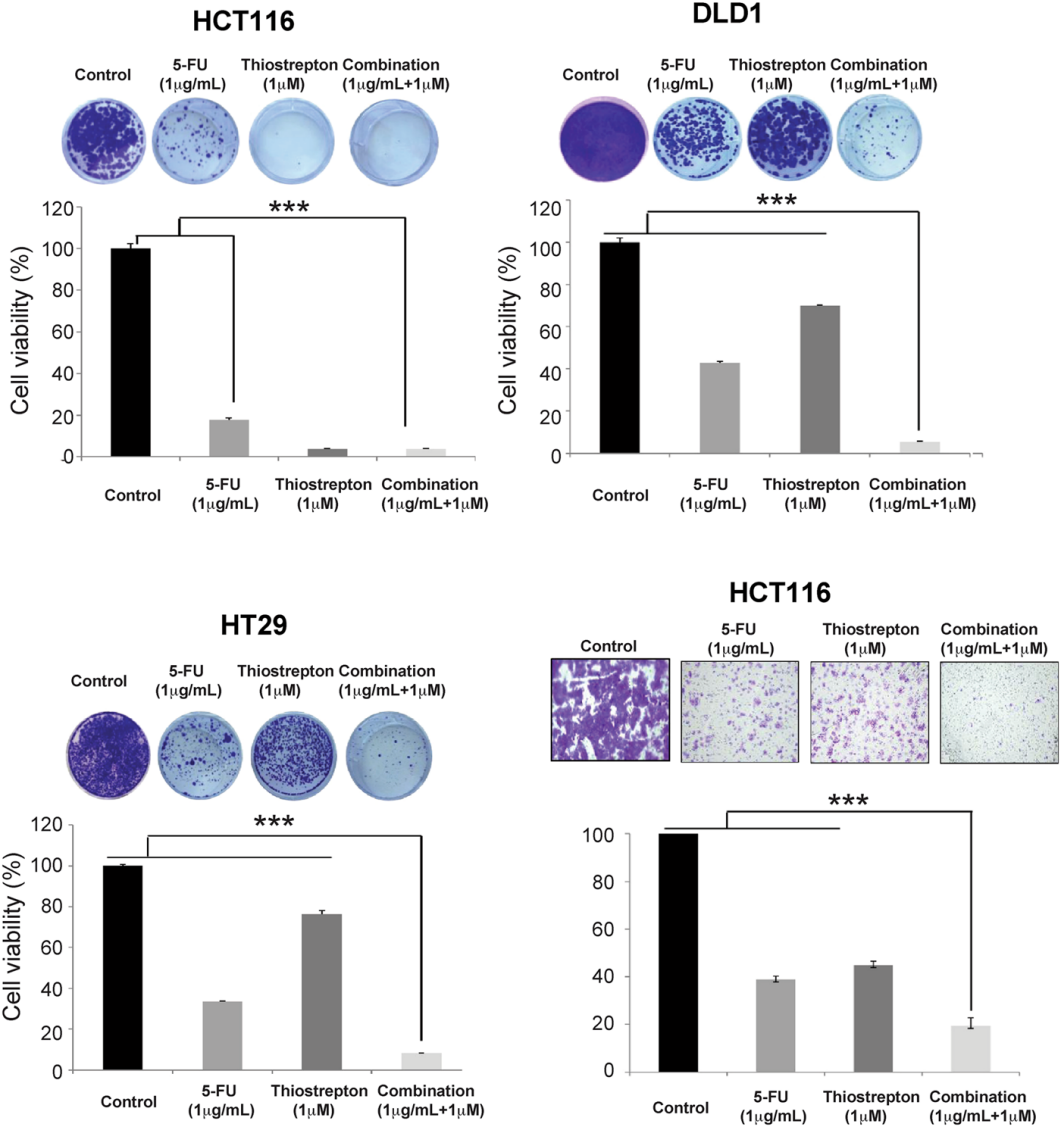
Combination treatment significantly inhibits tumour colony formation. (**A**–**C**) Clonogenic assays showing HCT116, DLD1 and HT29 cells lines treated with 5-FU (1 μg/ml) and thiostrepton (Thio) (1 μM) alone or in combination for 72 hours, followed by media replacement and culture for 14 days. The combination of 5-FU and thiostrepton significantly decreases colony formation in HCT116, DLD1 and HT29 cells. Data were represented as mean ± SD (n = 3). ***p < 0.0001. (**D**) 5-FU and thiostrepton combination significantly decreases migration of colorectal cancer cells, HCT116. Data were represented as mean ± SD (n = 2) (5-FU with combination p < 0.0001, thiostrepton with combination p < 0.0001).

Even at low doses of thiostrepton (1 μM) and 5-FU (1 μg/ml), marked inhibition of colony formation was observed in all 3 CRC cell lines, Fig. 7(A–C). Tumour cell colony formation was potently inhibited by the combination of 5-FU and thiostrepton when compared toeither agent given individually, and this effect was most notable for the p53 mutant DLD1 and HT29 cells. The combination treatment in HCT116 cells significantly inhibited tumour cell migration (3%) when compared with 5-FU (12%) alone and thiostrepton (22%) alone (Fig. 7D).

**Figure 8:**
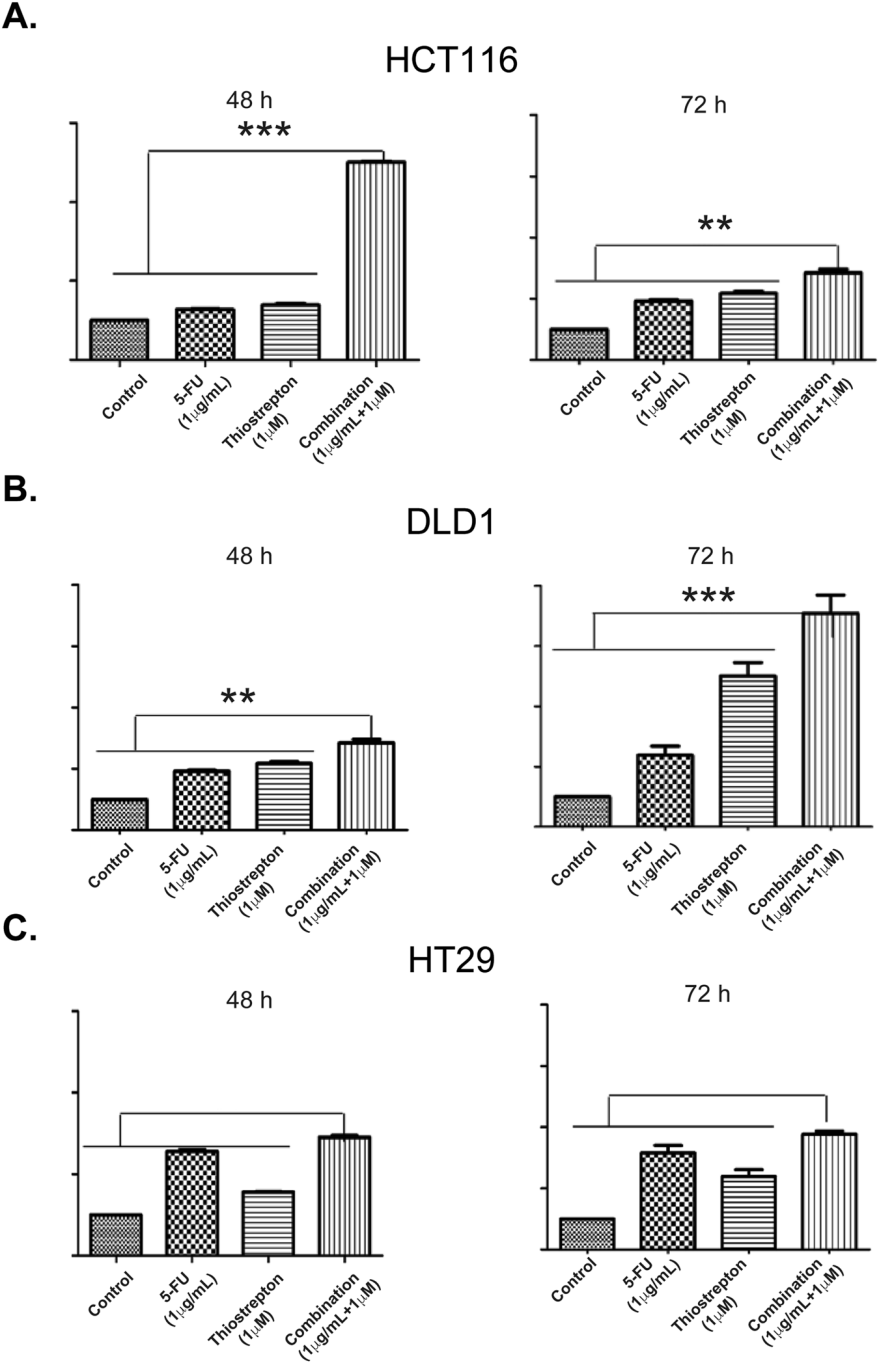
Combination treatment induces caspase-dependent apoptosis in colon cancer cells. Cellular caspase 3/7 activity measured in HCT116, DLD1 and HT29 cells following treatment with 5-FU (1 μg/ml) and thiostrepton (1 μM) alone or in combination for 48 and 72 hours. Data were represented as mean ± SD (n = 3). (**A**) 5-FU and thiostrepton combination significantly increased apoptosis compared to either agent alone or control HCT116 48 hours (p < 0.0001); HCT116 72 hours (p < 0.006). (**B**) DLD1 48 hours (p < 0.006); 72 hours (p < 0.0001). (**C**) HT29 48 hours (p < 0.005); 72 hours (p < 0.0001).

In HCT116 cells, combination treatment significantly increased caspase 3/7 activity at 24 hours and no changes observed in DLD1 or HT29 cells (Supplementary Fig. S8). However, at later time points (48, and 72 hours) lower levels of activated caspase3/7 wereobserved in HCT116 cells (Fig. 8A), which could be due to cell death in HCT116 cells after prolonged drug exposure. In DLD1 (Fig. 8B) and HT29 (Fig. 8C) cells, the combination treatment induced caspase-dependent apoptosis at 48 and 72 hours, but higher caspase levels observed at 72 hours. Even at low thiostrepton concentrations, caspase-dependent apoptosis was evident in p53 mutant DLD1 and HT29 cells in the combination treatment,

## Discussion

The FOXM1 transcription factor plays vital roles in cancer initiation, progression, metastasis, DNA damage repair, and resistance to apoptosis^31^. Our approach here was to determine if FOXM1 has a role in 5-FU resistance and if FOXM1 controls the expression of TYMS, a crucial target of anti-pyrimidines, commonly used to treat CRC. This is the first time that the function of FOXM1 in 5-FU resistance has been explored in CRC.

Initially, we profiled the expression of FOXM1 and TYMS in colorectal cancer cell lines, and also measured the expression of E2F1 as it is known to regulate TYMS and FOXM1 expression, independently^17–19^. Interestingly, with 5-FU treatment, we found similar expression patterns for FOXM1, TYMS and E2F1 protein and mRNA, potentially indicating a positive association between the expression of these genes. In p53 wt HCT116 cells, an initial induction of FOXM1, TYMS, and E2F1 expression was observed followed by a decrease at later time-points; however in p53 mutant DLD1 and HT29 colorectal cancer cells these proteins remain elevated, which is consistent with previous studies demonstrating that p53 is required for the downregulation of FOXM1 acting through p21Cip1^25^ and DNA-damage strongly up-regulated the level of FOXM1 in the absence of functional p53^19,25^. Our results also support previous studies showing that loss of functional p53 reduces cellular sensitivity to 5-FU^16^.

To investigate the functional correlations between the two proteins, we investigated the role of FOXM1 in 5-FU resistance in colorectal cancer cell lines. In our study, modulation of FOXM1 by transient transfection enhanced the resistance of HCT116 cells to 5-FU. Moreover, FOXM1 protein levels were higher in the acquired 5-FU resistant HCT116 cells compared to parental HCT116 cells. In addition, following 5-FU treatment, FOXM1 was ultimately down-regulated in HCT116 wt cells, whereas in the acquired 5-FU resistant HCT116 cells, persistent up-regulation of FOXM1 and TYMS protein expression was observed. This result suggests that elevated FOXM1 and concomitant TYMS expression is associated with 5-FU drug resistance, further demonstrating the crucial role of FOXM1 in 5-FU drug resistance and that the potential underlying mechanism is via modulation of TYMS expression. To confirm the importance of FOXM1 in TYMS and 5-FU resistance, we depleted TYMS in DLD1, HT29 and HCT116 pcDNA3 FOXM1 cell lines. Our results suggested that depleting TYMS alone in FOXM1 overexpressing cells may not influence 5-FU sensitivity (Supplementary Fig. S3), suggesting FOXM1 also regulates other targets to modulate 5-FU resistance.

In addition, inhibition of FOXM1 with thiostrepton significantly inhibited TYMS expression in both p53 wt and mutant cells. This result supports the theory that, downregulation of FOXM1 increases drug sensitivity in cancer cells, even in those with mutated p53^25^. Expression of E2F1 was not affected by FOXM1 overexpressing HCT116 cells, (Fig. 2C,D) but in thiostrepton treated HCT116 and HT29 colon cancer cells,(Fig. 3B,C) E2F1 was downregulated, suggesting that E2F1 is regulated by FOXM1.

Moreover, acquired 5-FU resistant HCT116 cells demonstrated the same IC50 as the parental HCT116 cells when cultured with thiostrepton. This could demonstrate that thiostrepton mediated inactivation of FOXM1 can help to reverse 5-FU resistance and targeting FOXM1 could potentially be a useful therapeutic strategy for overcoming 5-FU resistance (Supplementary Fig. S2). ChIP assay, (Fig. 4) also showed strong enrichment of FOXM1 occupancies on the TYMS promoter region, again supporting the significance of FOXM1 mediated 5-FU resistance through TYMS. FOXM1 inhibition by thiostrepton significantly decreased FOXM1 enrichment in TYMS promoter, further proving that TYMS is a direct target of FOXM1. Our ChIP-seq data confirmed FOXM1 has binding site on all the E2F transcription activators and FOXM1 regulates other cell cycle genes involved in RB/E2F1 pathways (Supplementary Fig. S5). This shows that even though E2F1 has a binding site on FOXM1 promoter^19^, E2F1 can down-regulated by the inhibition of FOXM1 (Fig. 3B,C). Further E2F1-3 siRNA knockdown experiments also reveal a complex co-regulatory network exists between E2F1-3, FOXM1, TYMS and TK-1 (Supplementary Figs S9 and S10).

FOXM1 binding was detected in the promoter regions of two other 5-FU targets: thymidine phosphorylase (TYMP) and thymidine kinase 1 (TK-1), suggesting that FOXM1 has a central role in the control of thymidylate biosynthesis and 5-FU resistance. In addition to that, Cyclin B1, Cyclin D1, CDKN1A (p21^Cip^), and DNA damage repair genes, RAD51, RAD54, XRCC1, BRCA2 and matrix metalloproteinases (involved in progression and metastasis) MMP9 and MMP2 were found to be regulated by FOXM1 in our ChIP-seq data. Consistent with our finding that FOXM1 has a vital role in 5-FU resistance, a recent study has shown that FOXM1 can enhance 5-FU resistance through promoting the efflux of antitumour drugs by up-regulating the ABC subfamily C member 10 (ABCC10) expression in colorectal cancer cells^32^. This mechanism will no doubt contribute to the global role of FOXM1 in mediating 5-FU resistance; however, it is also notable from our work that upon 5-FU treatment (1 µg/mL) (Fig. 2E), all the TYMS proteins were converted into the inactive and slower migrating FdUMP-ligated forms in the sensitive HCT116 colon carcinoma cell lines, whereas in the resistant HCT116 5-FU Res cells a great proportion of the overexpressed TYMS proteins remained active and uncomplexed^33^. This observation indicates that 5-FU has not been excluded by efflux and is able to enter the nuclei of the cancer cells and complex with TYMS in the 5-FU resistant cells, and also that the upregulation of TYMS associated with FOXM1 overexpression in the resistant colon carcinoma cells plays a predominant role in mediating 5-FU insensitivity through upregulation of the drug targets.

We also identified a synergistic relationship between thiostrepton and 5-FU in colorectal cancer cells. Our study demonstrated that compared with 5-FU and thiostrepton alone, the combination treatment significantly increased ɣH2AX foci. This indicates that thiostrepton enhances 5-FU mediated DNA damage by inhibiting FOXM1 and its genomic targets involved in DNA repair pathway. This finding is in agreement with other studies showing that higher levels of γH2AX staining in *Foxm1*^−/−^ MEFs compared with WT MEFs upon epirubicin treatment. This study also identifies BRIP1 as a direct transcription target of FOXM1, which is an important mediator for DSB repair^34^. Another study from the same lab shows that FOXM1 regulates the expression of NBS1, which involved in homologous recombination (HR) DNA repair activity. Our studies also demonstrated that combined treatment of thiostrepton and 5-FU decreased colony formation and migration ability in colon cancer cells, and also induced caspase 3/7 dependent apoptosis even in p53 mutant colon cancer cells, supporting previous studies that showed proteasome inhibitors like thiostrepton can induce p53-independent apoptosis in human cancer cells^35–37^.

FOXM1 is becoming recognised as an important target for cancer therapeutics, and its increased expression has been documented in many diverse tumour types, including colon, breast, prostate, and cervical cancers^31^. In normal colonic mucosa, FOXM1 expression is weak; however strong staining was observed in the matched primary tumours and notably this was more pronounced in the nodal metastases of the same patients^38^. In CRC, Li *et al*. also found that FOXM1 binds specifically to two regions of the PLAUR (urokinase-type plasminogen activator receptor) gene promoter, and it gene product functions to degrade the extracellular matrix during cell migration and proliferation, supporting the view that FOXM1 is a master regulator of tumorigenesis^38^. Significantly, in that cohort of patients, high FOXM1 expression was found to be an independent prognostic factor associated with a worse 5 year overall survival (45.6 versus 85.9%, HR 3.62), and a 4 fold increased risk of metastasis. Future studies should involve a larger cohort of tumour samples with survival data used to validate FOXM1 as a biomarker for drug resistance.

To conclude, in this study, we have demonstrated for the first time that FOXM1 has a crucial role in 5-FU drug resistance in colon cancer cells, through regulating some of the main 5-FU targets, including TYMS, TK-1 and TYMP. Our study also has also shown that thiostrepton can exert synergistic effects with 5-FU and that combination treatment decreases cell viability, migration, and increases DNA damage and apoptosis in colon cancer cells. Thiostrepton can downregulate FOXM1 expression and induce cytotoxicity in p53 mutant and acquired 5-FU Res cell lines. Our data reveal the benefit of thiostrepton in combination with 5-FU treatment, which could potentially allow us to target the p53 mutant and acquired drug resistant cancers. In addition, FOXM1 could also be a novel predictive biomarker in 5-FU therapy, and new treatment strategies that target FOXM1 should be developed to improve clinical response in CRC.

## Materials and Methods

### Cell culture

All cell lines were authenticated by short tandem repeat profiling (DNA diagnostic centre, London, UK) and were confirmed to be Mycoplasma-negative. Human colorectal cancer cell line HT29 (DSMZ Braunschweig, Germany), were maintained in DMEM (Dulbecco’s Modified Eagle’s Medium)(Sigma) supplemented with 10% Foetal Calf Serum, 200 mM L-glutamine, penicillin/streptomycin in a humidified incubator with 5% CO2 at 37 °C. HCT116 and DLD1 cells were maintained in RPMI-1640 (Sigma), supplemented with 10% Foetal Calf Serum, 200 mM L-glutamine, penicillin/streptomycin in a humidified incubator with 5% CO2 at 37 °C. Acquired 5-FU resistant HCT116 cells were a gift from Dr Longley/Professor Johnston’s laboratory, Queen’s University Belfast, and maintained as previously described^39^. 5-FU (F6627-10G) and thiostrepton (T8902) were purchased from Sigma, Poole, UK and were dissolved in dimethylsulphoxide.

### Western blotting

Western blotting was performed on whole cell extracts by lysing cells in buffer as previously described^40^. Antibodies against FOXM1 (C20) cat no: SC502, E2F1 (C20) cat no: SC193, and β-tubulin (H235) cat no: SC9104, were purchased from Santa Cruz Biotechnology, Inc. TYMS ab58287, TK-1 ab57757 were purchased from Abcam. Primary antibodies were prepared in bovine serum albumin (BSA) in 1:1000 dilution based on manufacturers’ instructions and detected by using the appropriate anti-mouse and anti-rabbit conjugate (DAKO), prepared in TBST in 1:2000 dilution and visualized using the enhanced chemiluminescence detection system (ECL) (Perkin Elmer).

### Sulforhodamine B (SRB) Assay

3,000–5,000 cells were seeded in each well of the 96-well plates. After 24 hours cells were treated with various concentrations of each drug as indicated in. After 72 hours drug exposure, SRB assay were conducted as described^40^. Optical density at 540 nm was measured in a microplate reader.

### Transfection

For gene silencing, cells were transiently transfected with 20 nM of ON-TARGET plus SMARTpool siRNAs (Dharmacon). Transfections were performed using oligofectamine reagent (Invitrogen) following the manufacturer’s instructions.

The SMARTpool siRNAs used were: FOXM1 (L-009762-00), TYMS (E-004717-01-0005) and non-specific siRNA control (NSC) (D-001810-10-20-00). Prior to siRNA transfection, cells were grown in appropriate culture dishes until they reached 50–60% confluence. 30,000 cells per well of a 6-well plate were plated 24 h prior to transfection. Following the manufacturer’s protocol, cells were harvested, for western blotting and mRNA expression or the transfected cells were treated with cytotoxic agents and SRB assays was performed to detect cell viability. For transient overexpression pcDNA3 FOXM1, transfections were performed using X-treme GENE HP (Roche).

### Real-time Quantitative PCR

Total RNA was extracted using the RNeasy Mini Kit (Qiagen), and cDNA was prepared using the superscript III reverse transcriptase and oligo-dT primers (Invitrogen). For real-time quantitative PCR (RTQ-PCR), 100 ng cDNA was added to SYBR green master mix (Applied Biosystems) and run in 7900 HT Fast Real-time PCR System (Applied Biosystems). The cycling program was 95 °C for 10 min followed by 40 cycles of 95 °C for 30 s and 60 °C for 30 min. Each sample was assayed in triplicate. Transcript levels were quantified using the standard curve method. L19, a non-regulated ribosomal housekeeping gene was used as an internal control to normalize input complementary DNA.

The forward and reverse primers used were as follows,

L19-F 5′ GCGGAAGGGTACAGCCAAT,

L19-R 3′ GCAGCCGGCGCAAA,

FOXM1-F 5′ TGCAGCTAGGGATGTGAATCTTC,

FOXM1- R 3′ GGAGCCCAGTCCATCAGAACT,

TYMS- F 5′ GCCTCGGTGTGCCTTTCA

TYMS-R 3′ GATGTGCGCAATCATGTACGT

E2F1-F 5′ ATGTTTTCCTGTGCCCTGAG,

E2F1-R 3′ ATCTGTGGTGAGGGATGAGG

### Immunohistochemistry staining

Human CRC tissue microarray samples containing 110 tumours were purchased from Biomax USA (product number BC051110). FOXM1 and TS immunoreactivity was evaluated on TMA sections using polyclonal rabbit anti-human FOXM1 (C20) antibody (Santa Cruz Biotechnology) and TS antibody (Abcam) in a modification of the antigen retrieval technique of Shi *et al*.^41^. Briefly, the sections were rehydrated and heated in a microwave oven at 900 W for 20 min in citrate buffer at pH6. Primary antibodies were used at 1:100 (FOXM1) and 1:150 (TS) and 1:150 (TK-1) dilution for 1 hour at room temperature and then processed with Polymer-HRP Kit (BioGenex, San Ramon CA) with Diaminobenzidine development and Mayer haematoxylin counterstaining. External positive controls were used as suggested by the company. Negative controls were obtained by omitting the primary antibody. A semi-quantitative immunohistochemical score (IHS) was calculated by an independent accredited histopathologist using the intensity of staining and the percentage of positive cells as previously described^42^. The scoring was calculated based on percentage of positive cells and intensity of the staining in TYMS Cytoplasm, TK-1 cytoplasm and nucleus, FOXM1 cytoplasm and nucleus. I have only scored TYMS cytoplasm, based on previously published IHC data and TYMS immunoreactivity was demonstrated mainly in the cytoplasm of cancer cells. The following scoring system was used: The staining intensity (0, 1, 2, and 3) and the fraction of positive tumour cells were recorded for each tissue spot. Negative scores had staining intensity of 0 (tissues missing or no staingn), weak scores had percentage of positive cells in 10–40%, moderate score had percentage of positive cells in 40–70% and high score had percentage of positive cells in 70–100% of tumour cells. Two values were multiplied and calculated the score.

### Chromatin Immunoprecipitation (ChIP)

HCT116 and DLD1 cells were grown to 90–95% confluency (150 mm dish) and cross-linked with 1% formaldehyde for 10 min at room temperature, 125 mM of glycine was added for 5 min to stop cross-linking. The cells were washed with cold PBS and harvested using the cell lysis buffer with 1X PI (protease inhibitor). Following sonication, (10 min for HCT116 and 12 min for DLD1) the samples were cleared by centrifugation (14,000 g/10 min/4 °C), and supernatants were transferred into new tubes. Antibody binding (FOXM1 C-20×, Santa Cruz biotechnology) was done using IgG protein-A and IgG protein-G dynabeads (Invitrogen) by incubating in a rotator for overnight in 4 °C. The dynabeads were then washed with TSE I + PI. DNA was eluted using elution buffer and decross linked the samples overnight at 650 C or 6 hours. Extracted the DNA with Qiagen PCR purification kit and ran the samples in 7900 HT Fast Real-time PCR System (Applied Biosystems). FOXM1 binding was analysed by RT-PCR, and normalised to the input sample. Data were presented as a fold enrichment relative to control primers that were designed from 2000–4000 bp length.

Primers used for ChIP were 0 to −500 bp

FW_CCTGGCGGTTTTTAATCAAG R_CACAGTTCCCACGTTTTCCT

FW_CCTGGCGGTTTTTAATCAAG R_ACAGTTCCCACGTTTTCCTG

−500 to −1000 bp

FW_GTAGTCCCAGCTACGCGAGA R_AGGGCTTTTCCGAGGTTTT

−1000 to −1500 bp

FW_CTGCTGAGGGCTCTATCACC R_CTCGGCCCCAAGTTTTTAAT

−1500 to −2000 bp

FW_GACCACTCCTCTGGGTCAAA R_ACCTGTCTTGAGGCCTGTGT

FW_TTCTACGCCCAGAACCATCT R_GGCCTGTGTTGATGTCAATG

−2000 to −3000 bp

FW_GGCACAGGAAGAAAGGTCTG R_TTGTGGTGCTTGCTGTACTTG

−2000 to −3000 bp

FW_GGGCACAGGAAGAAAGGTCT R_GTGGTGCTTGCTGTACTTGG

−3000 to −4000 bp

FW_GGCACAGGAAGAAAGGTCTG R_CCTTGTGGTGCTTGCTGTACT

### ChIP-Seq

The ChIP-ed DNA and input DNA were amplified as described above. Libraries were generated, and sequencing was performed on Illumina Hi Seq2000 analyser according to the manufacturer’s protocol. IGV (interactive genome viewer)^43^ was used to visualise ChIP sequenced data. The basic bed file format was used to visualise ChIP-seq peaks. The Cis-regulatory Annotation System (CEAS) function in Cistrome was used to functionally annotate binding sites^44,45^.

### Immunofluorescence

Exponentially growing HCT116 and HCT116 5-FU res cells were seeded in chamber slides on day 1. The cells were treated on day 2 with 1 µg/ml 5-FU and 1 µM thiostrepton and combination of both drug for 24 h. Cells were washed in PBS and then incubated in 2% paraformaldehyde in PBS for 10 min, washed in PBS, permeabilized in 0.1% triton X100, diluted in PBS at room temperature (RT) for 10 min, washed, blocked with PBS containing 1% bovine serum albumin, and incubated overnight at 4 °C with an anti-phosphohistone H2AX antibody, washed with PBS and incubate secondary antibodies: Alexa Fluor 594 (Red) and 488 (green) at 1:500 (Invitrogen). Cells were then washed 3 times with PBS and mounted with Vectashield mounting medium containing DAPI (Vector Laboratories Inc.). Slides were mounted and visualized with a Leitz Laborlux UV microscope (Wetzlar, Germany) using a ×40 objective fitted with a Spot Insight 4 camera (Diagnostic Instruments, Sterling Heights, Michigan). The intensity of fluorescence was evaluated with ImageJ software.

### Clonogenic assay

Tumour colony formation assay was conducted in 6 well plates. 2000–3000 cells/well were seeded, after 24 hours cells were treated with 5-FU (1 µg/mL), thiostrepton (1 µM) alone, or in combination for 72 hours. After 72 hours drug free medium was added and the cells were grown for 12–14 days (with media changes every 3 days), colonies were fixed with 4% formaldehyde, and stained with crystal violet (0.05%). Stained plates were photographed, and then the colonies were dissolved with 15% acetic acid. Optical density at 540 nm was measured in a microplate reader (Sunrise, Tecan).

### Migration assay

HCT116 cells were seeded in 6 well plates and treated with 5-FU (1ug/mL), thiostrepton (1 µM) alone or combination 5-FU and thiostrepton at the same concentration for 48 hours. The cells were then harvested and seeded to the upper chamber of the inserts (10000 cells/well in 200 ul of serum free RPMI). 750 ul of RPMI medium with 10% FBS was added to the lower chamber. The cells were allowed to migrate for 48 hours at 37 °C. The cells were removed from the upper side of the transwell with a cotton swab. The invading cells on the underside of the transwell were fixed with 4% formaldehyde and stained with crystal violet. The stained invasive cells were photographed under a microscope.

### Caspase 3/7 activity

Caspase 3/7 dependent apoptosis was measured by using a Promega Caspase-Glo 3/7 assay. Briefly, 5000–6000 cells were seeded in white walled 96 well plate. After 24 hours cells were treated with 5-FU (1 µg/mL), thiostrepton (1 µM) alone or in combination for 72 hours. Then 50 μl of Caspase-Glo 3/7 reagent was added to each well and the contents of wells were mixed using a plate shaker at 300–500 rpm for 1 minute, followed by incubation at room temperature for 60 minutes. The luminescence of each sample was measured in a plate-reading luminometer.

### Statistical Analysis

IC50 values were calculated using GraphPad Prism software and the best-fit sigmoidal curve was applied to the data, paired t-tests were used to compare differences between NSC and SiRNA or over-expressing cell lines. The correlation coefficient between tissue protein expressions in microarrays was calculated using Spearman’s rho coefficient with SpSS software.

## Supplementary information

Supplementary Figure S1-S17
