# Supplementary material for: FOXM1 modulates 5-FU resistance in colorectal cancer through regulating TYMS expression
Source: Sci Rep. 2019 Feb 6;9:1505. doi: 10.1038/s41598-018-38017-0 (PMC6365533; doi:10.1038/s41598-018-38017-0)
Supplement: Supplementary file 1 — Supplementary Figure S1-S17 [file 41598_2018_38017_MOESM1_ESM.pdf]

Supplementary Figure S1-17 for the manuscript:

FOXM1 modulates 5-FU resistance in colorectal cancer through regulating TYMS expression.

Vidhya Varghese, Luca Magnani, Narumi Harada-Shoji, Francesco Mauri, Richard M Szydlo, Shang Yao , Eric W-F Lam and Laura M Kenny

Supplementary Figure S1

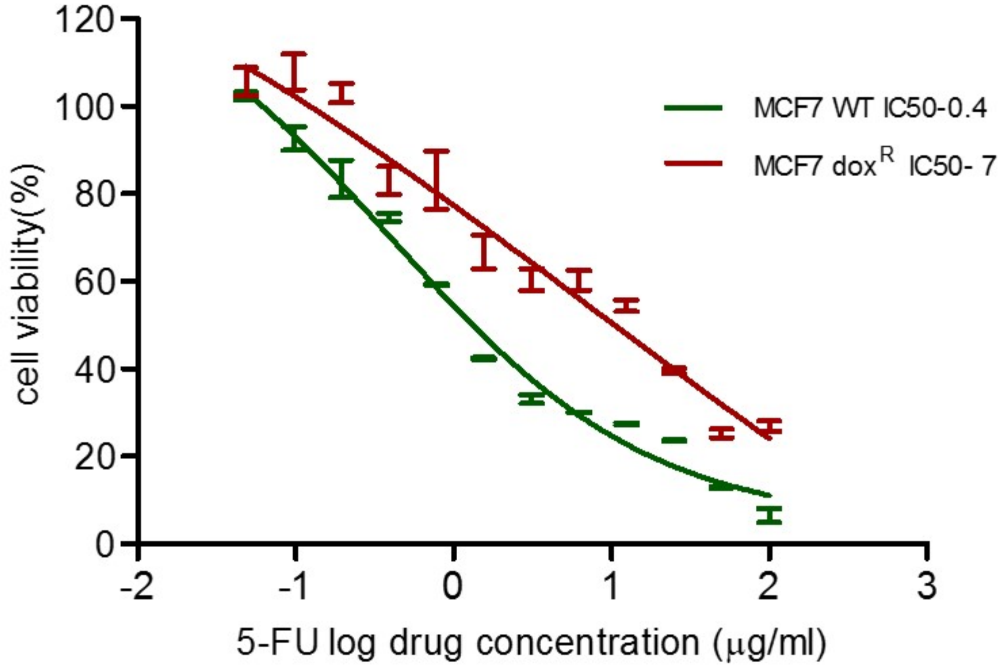

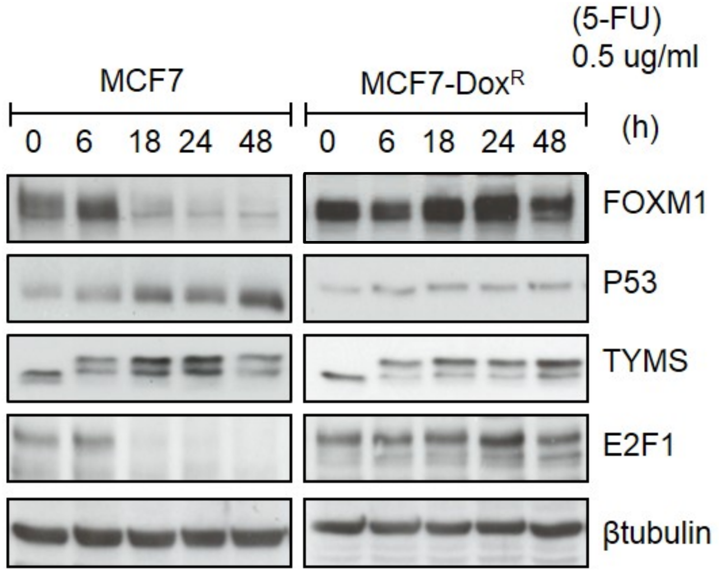

Supplementary figure S1.

MCF7 wt and MCF7 doxorubicin resistant cells were treated with 5-FU, ranging from 0 to 100 μg/ml for 72 h. IC50 values were: MCF7 0.4±0.3 and MCF7 DoxR 7±0.2. Results shown are mean and SD of two independent experiments, each with three replicates. IC50 values were determined by fitting a sigmoidal dose-response curve to the data using Graph pad Prism®. MCF7 wt and MCF7 doxorubicin resistant cells were treated with 0.5 μg/ml 5-FU for 0, 6, 18, 24, and 48 h. Protein expression was quantified using a standard curve and normalised to the housekeeping gene L19. In 5-FU treated MCF7 wt cells down regulation of FOXM1, E2F1, and TYMS and up regulation of P53 observed. But no significant changes observed in MCF7 DoxR cells.

Supplementary Figure S2

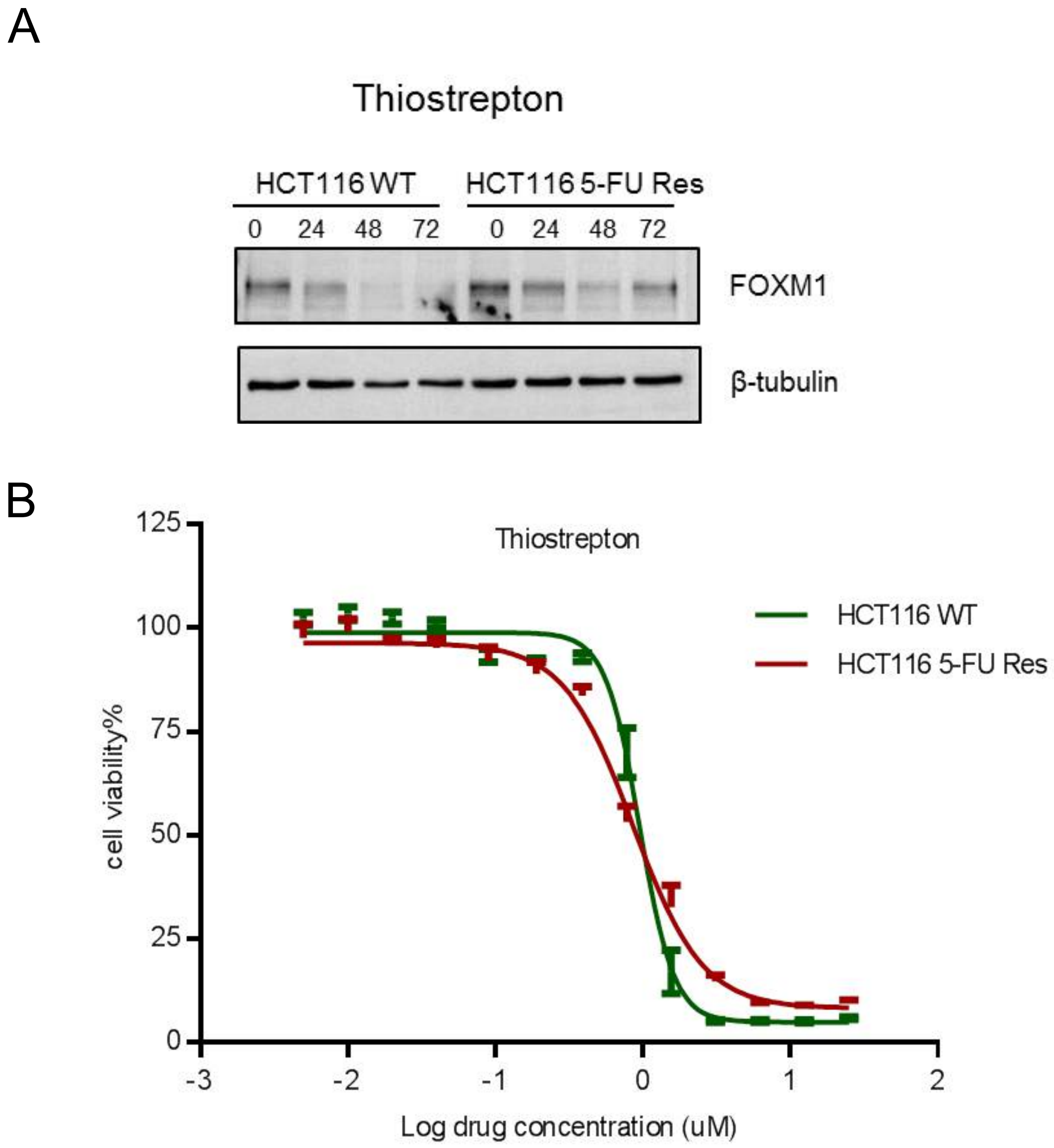

Supplementary Figure S2
Thiostrepton can alter 5-FU resistance through the inhibition of FOXM1. A. Western blots were carried out to detect the expression of FOXM1 after thiostrepton treatment. HCT116 wt, HCT116 5-FU res cells were treated with 2 μM of thiostrepton for 0, 24, 48 and 72h. B. HCT116 wt, HCT116 5-FU Res cells were treated with thiostrepton ranging from 0-200μM for 72h. SRB assays indicate that the treatment decreases the cell viability of HCT116 5-FU Res along and HCT116 wt cells (IC50 0.8 μM). Results were normalised to untreated. Results shown are mean and SD of three independent experiments, each with three replicates. IC50 values were determined by fitting a sigmoidal dose-response curve to the data.

Supplementary Figure S3

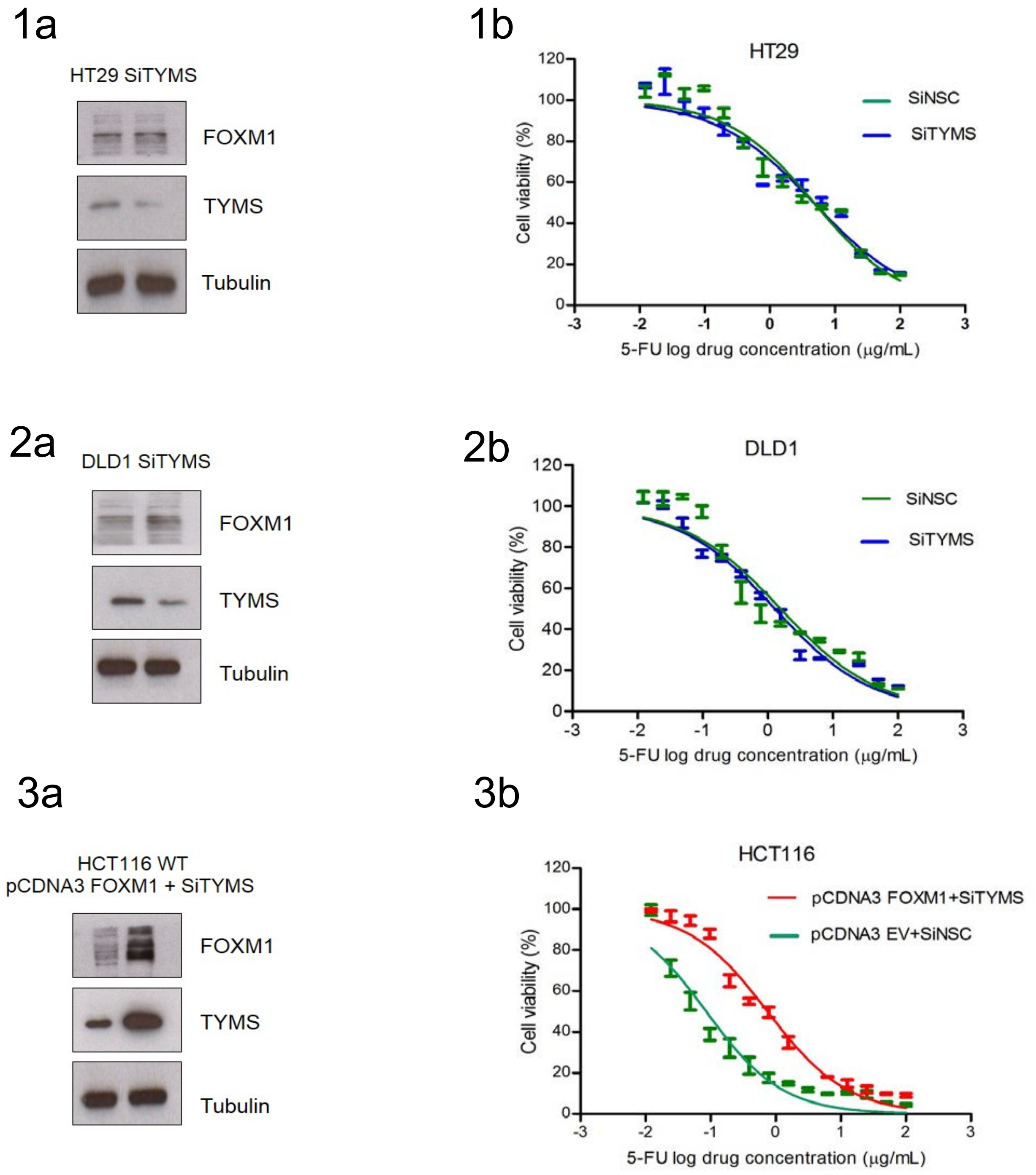

Supplementary Figure S3
HT29 (figure 1a), and DLD1 (figure 2a) cell lines were transiently transfected with SiTYMS. After 48 h transfection cells were harvested and probed for FOXM1 and TYMS by Western blotting. Further to TYMS knocked down, (Figure 1b, figure 2b) cells were treated with 5-FU, ranging from 0 to 100 µg/ml for 72 h and looked at the cell viability. No significant changes observed between control and SiTYMS in both HT29 and DLD1.
HCT116 (figure 3a), cells were co-transfected with pcDNA3 FOXM1 plasmid and SiTYMS and looked at protein expression of FOXM1 and TYMS. Western blotting confirmed that the over expression of FOXM1 and SiTYMS led to a downregulation of TYMS protein expression. Further to co-transfection, cells were treated with 5-FU, ranging from 0 to 100 µg/ml for 72 h (figure 3b). Overexpressed FOXM1 increased chemoresistance in HCT116 cells measured using SRB assays, whereas coexpression of SiTYMS has little effect.

Supplementary Sigure S4

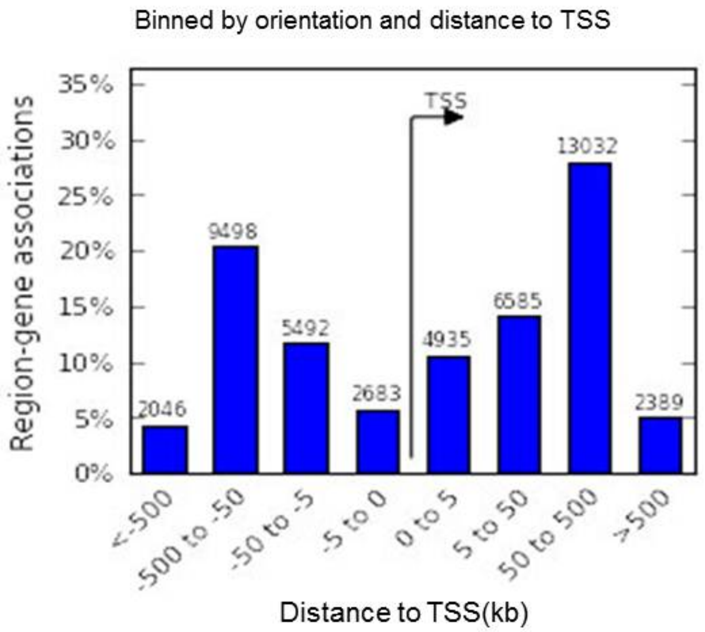

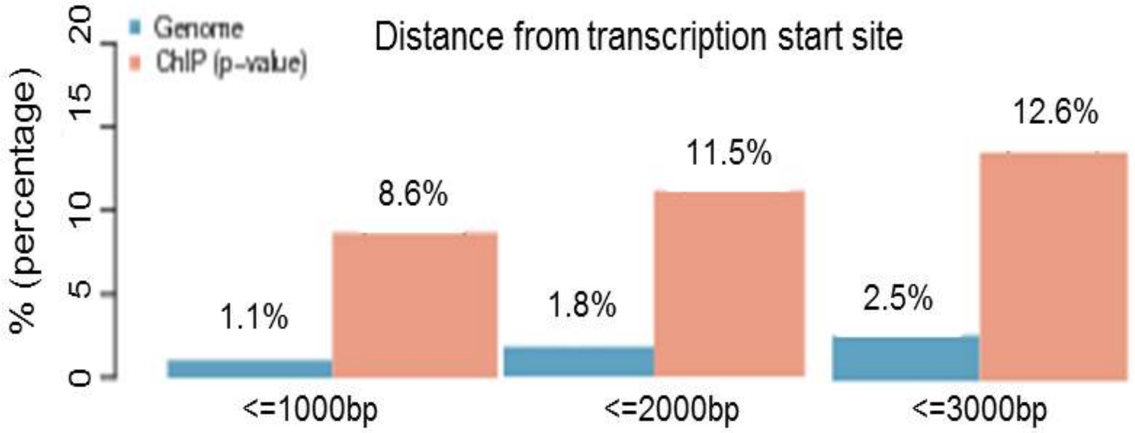

Supplementary Figure S 4
The distance to TSS graphs show the gap in base pairs between input regions and their putatively regulated genes, divided into four separate groups 0-5kb, 5-50kb, 50-500kb, and >50kb. Figure B. The bottom panel shows genomic distribution of FOXM1 binding events in 0 to 3000bp upstream in the promoter

Supplementary Figure S5

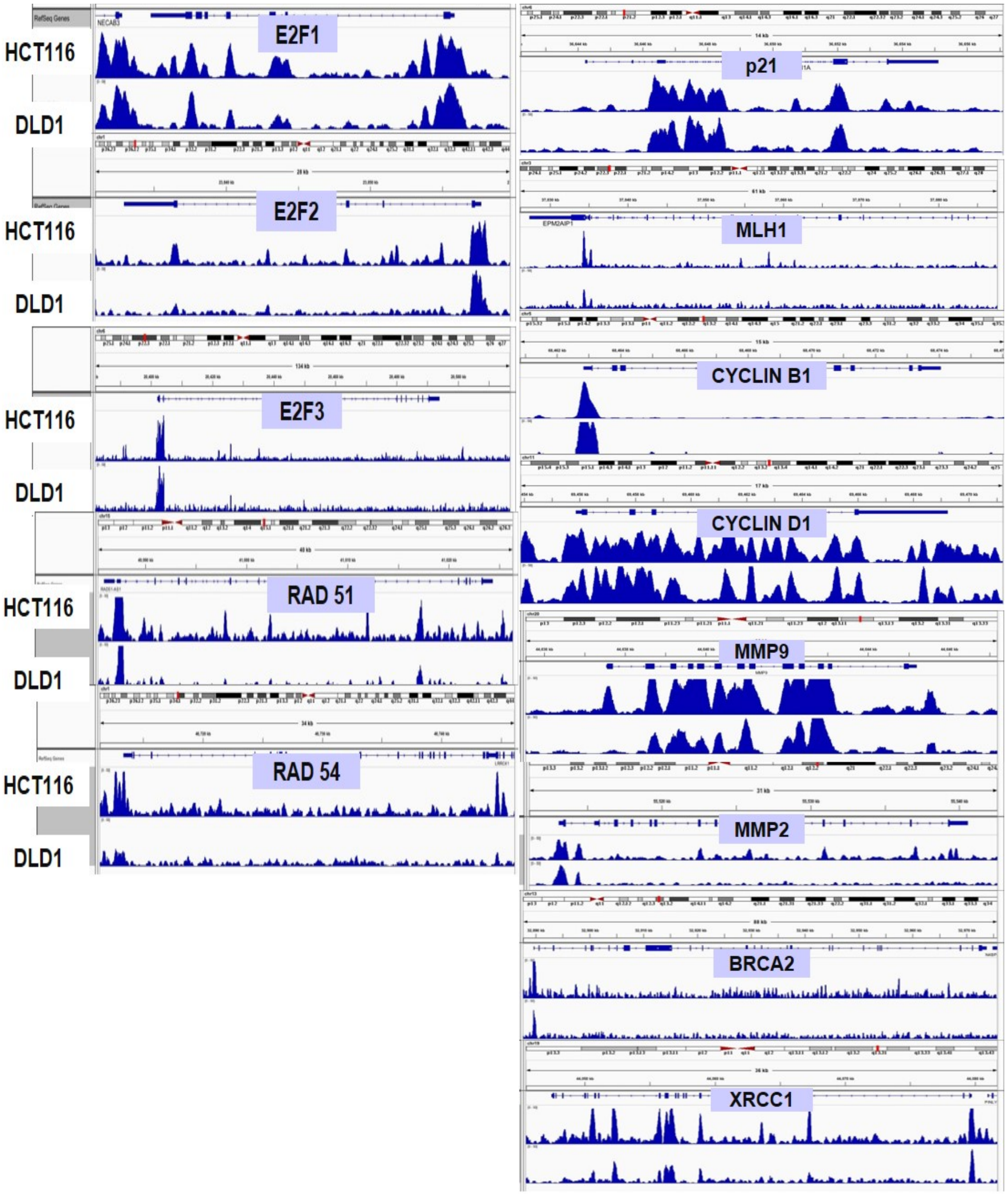

Supplementary Figure 5
The Integrative Genomics Viewer (IGV) browser was used to visualise the FOXM1 binding in down-stream targets. Cell cycle regulatory genes: Cyclins B1, Cyclins D1, CDKN1A (p21Cip), CDKN1C (p57). Mismatch repair gene: MLH1, DNA damage repair gene: RAD51, RAD54, XRCC1, BRCA2 and matrix metalloproteinase (Metastasis and progression): MMP9, MMP2 in HCT116 and DLD1 cells.

Supplementary Figure S6

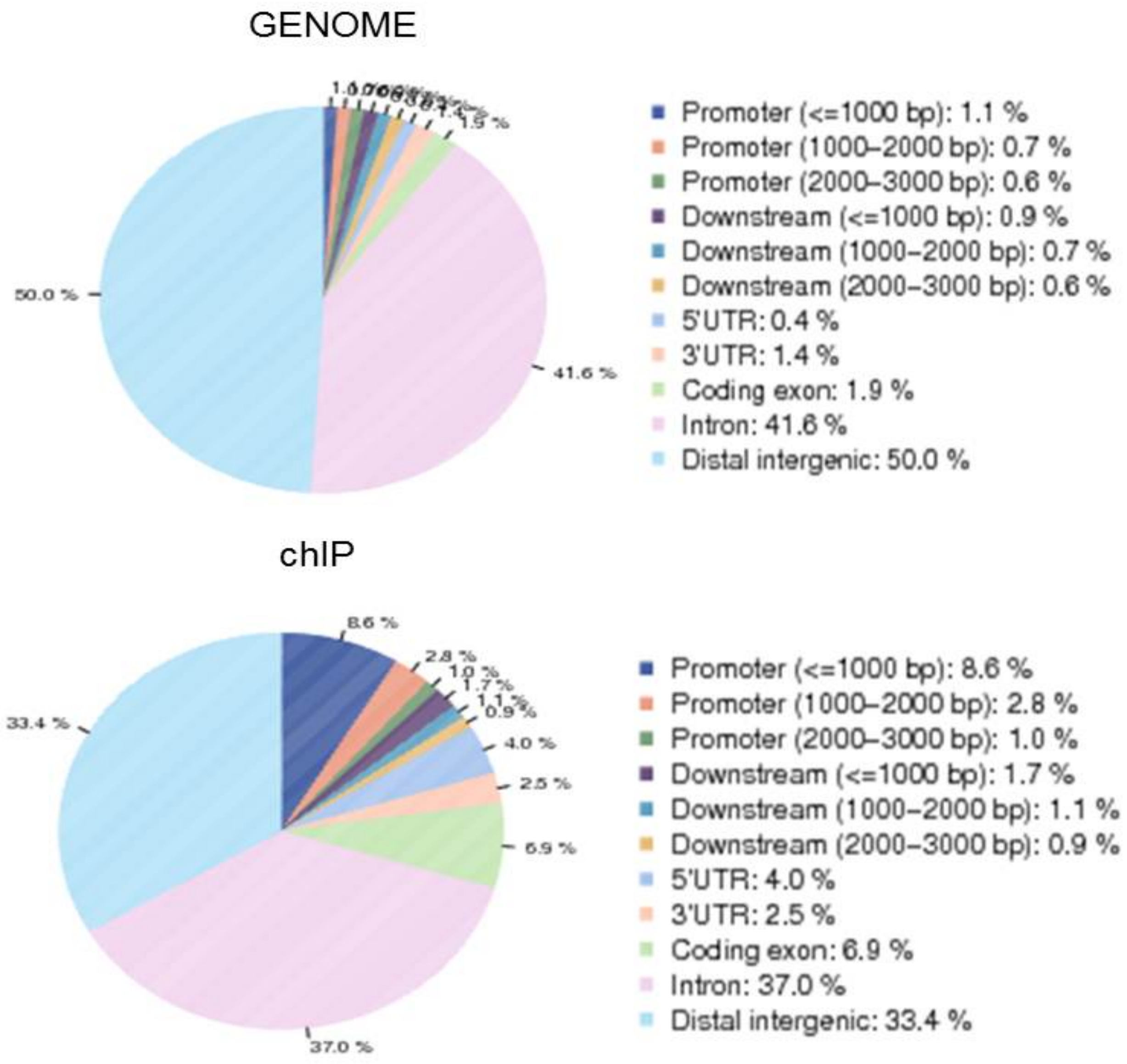

Supplementary Figure S6
CEAS analysis showing genomic distribution of FOXM1 binding events in HCT116 cells. Distribution of FOXM1 ChIP-seq regions (bottom) compared to the total genomic DNA distribution (top).

Supplementary Figure S7

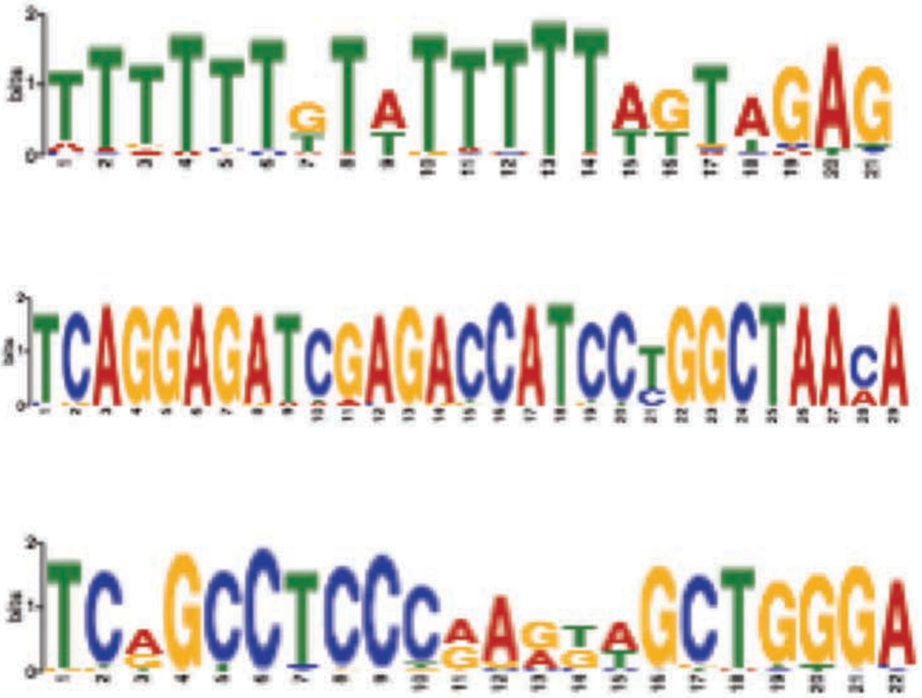

Supplementary Figure S7
Significantly enriched 3 top motifs logo were identified by MEME software (200bp regions around peak centre). From each

Supplementary Figure S8

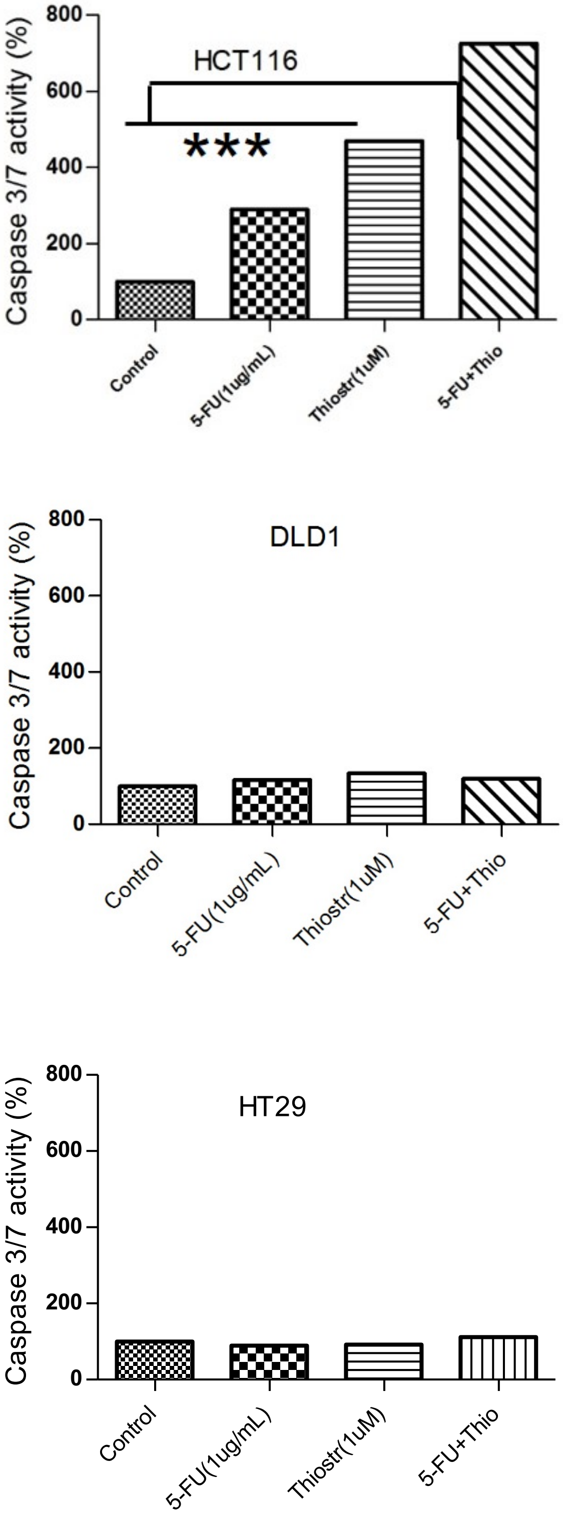

Supplementary Figure S8
Cellular caspase 3/7 activity measured in HCT116, DLD1 and HT29 cells followed by 5-FU (1**µ**g/ml) and thiostrepton (1 **µ**M) treatment alone or in combination for 24 hours. 5-FU and thiostrepton combination significantly increased apoptosis in HCT116 cells compared to either agent alone or control HCT116 ($p<0.0001$). But in DLD1 and HT29 cells no caspase 3/7 activity observed in drug alone or in combination for 24 hours.

Supplementary Figure S9

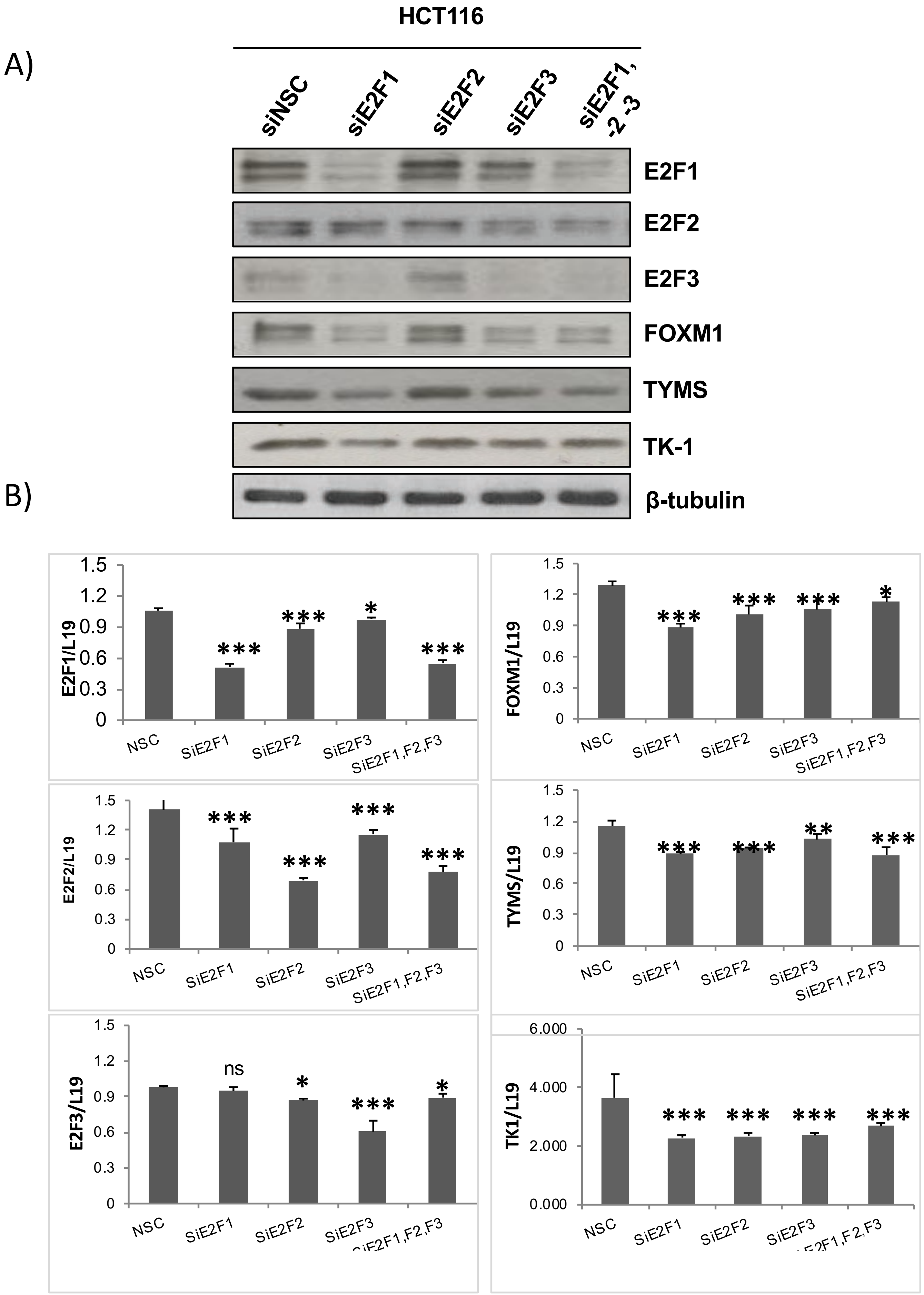

Supplementary Figure S9 (Cont'd)

Supplementary Figure 9

The contribution of E2F1-3 in regulation of FOXM1, TYMS and TK-1 in coloncarcinoma cells. HCT116 cells were transiently transfected with siE2F1, or siE2F2, or siE2F3, or combination of all three E2F siRNA pools. The controls were transfected with non-specific siRNA (NSC). For gene silencing, cells were transiently transfected with 20nM of ON-TARGET plus SMARTpool siRNAs (Dharmacon) against E2F1 (L-003259-00), E2F2 (L-003260-00), E2F3 (L-003261-00) and non-specific siRNA control (NSC) (D-001810-10-20-00). Transfections were performed using oligofectamine reagent (Invitrogen) following the manufacturer's instructions as described. Cells were harvested 48h post-transfection for Western blot and RT-qPCR analysis. A) Western blot analysis showed that knock-down of E2F1-3 was associated with decreases in the protein expression of FOXM1, TYMS and TK-1. B) RT-qPCR was also performed to investigate the mRNA levels of E2F1, E2F2, E2F3, TYMS, TK-1 and FOXM1 in HCT116 following E2F1, E2F2, E2F3 and E2F1-3 depletion using siRNA smart pools. Error Bars represent standard deviation. Statistical significance was determined by student's T-test. (*p value < 0.05, **p value < 0.01, ***p value < 0.05, versus control; ns=non significant). Depletion of E2F1, E2F2, E2F3 or E2F1-3 was associated with reductions in the mRNA expression of E2F1, E2F2, E2F3, TYMS, TK-1 and FOXM1. Triple knock-down of E2F1-3 transcription activators was not significantly superior to E2F1. -2, or -3 depletion in decreasing FOXM1, TYMS and TK-1 mRNA expression.

Supplementary Figure S10

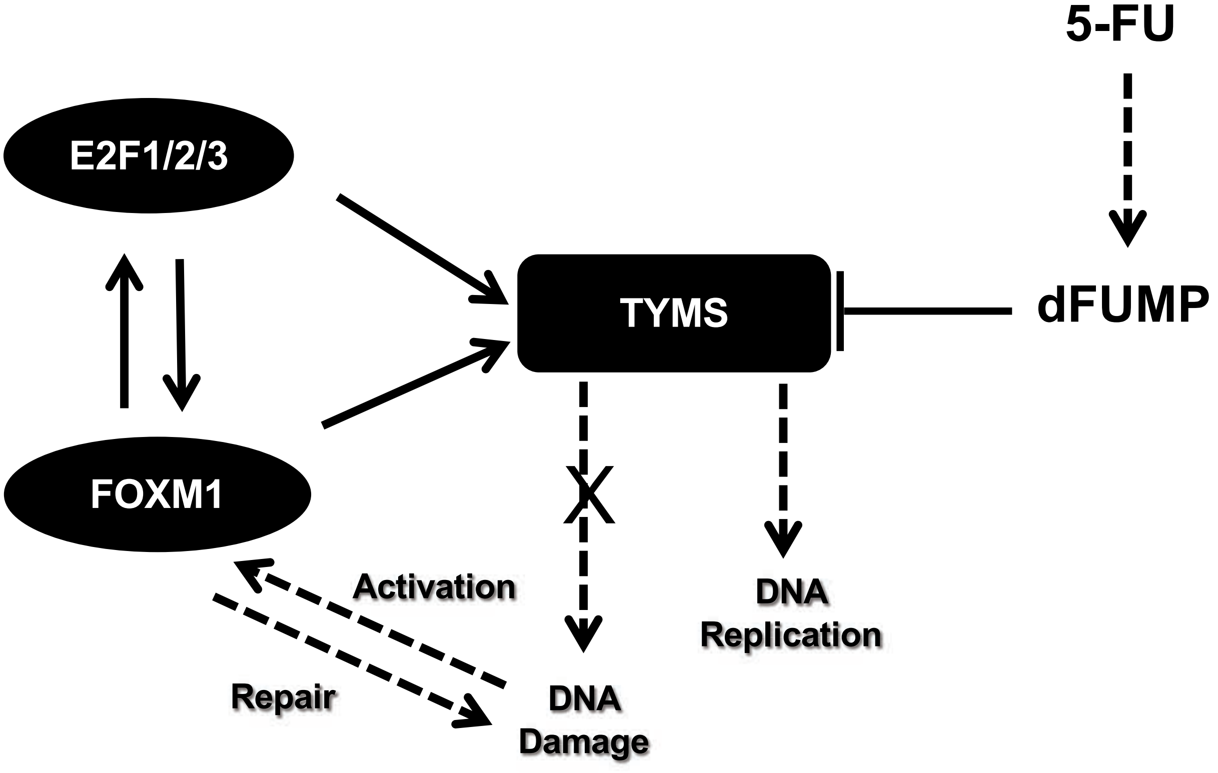

Supplementary Figure S10

Schematic diagramme of the proposed mechanism for the regulation of TYMS expression by FOXM1 and E2F1 in response to 5-FU. TYMS expression is regulated by both FOXM1 and E2F1 which also reciprocally regulate the expression of each another. FOXM1 regulates genes involved in DNA damage repair and therefore genotoxic drug resistance. FOXM1 expression and activity are activated by DNA damage, which can be triggered by 5-FU through its inhibition of the DNA replication function of TYMS.

Supplementary Figure S11

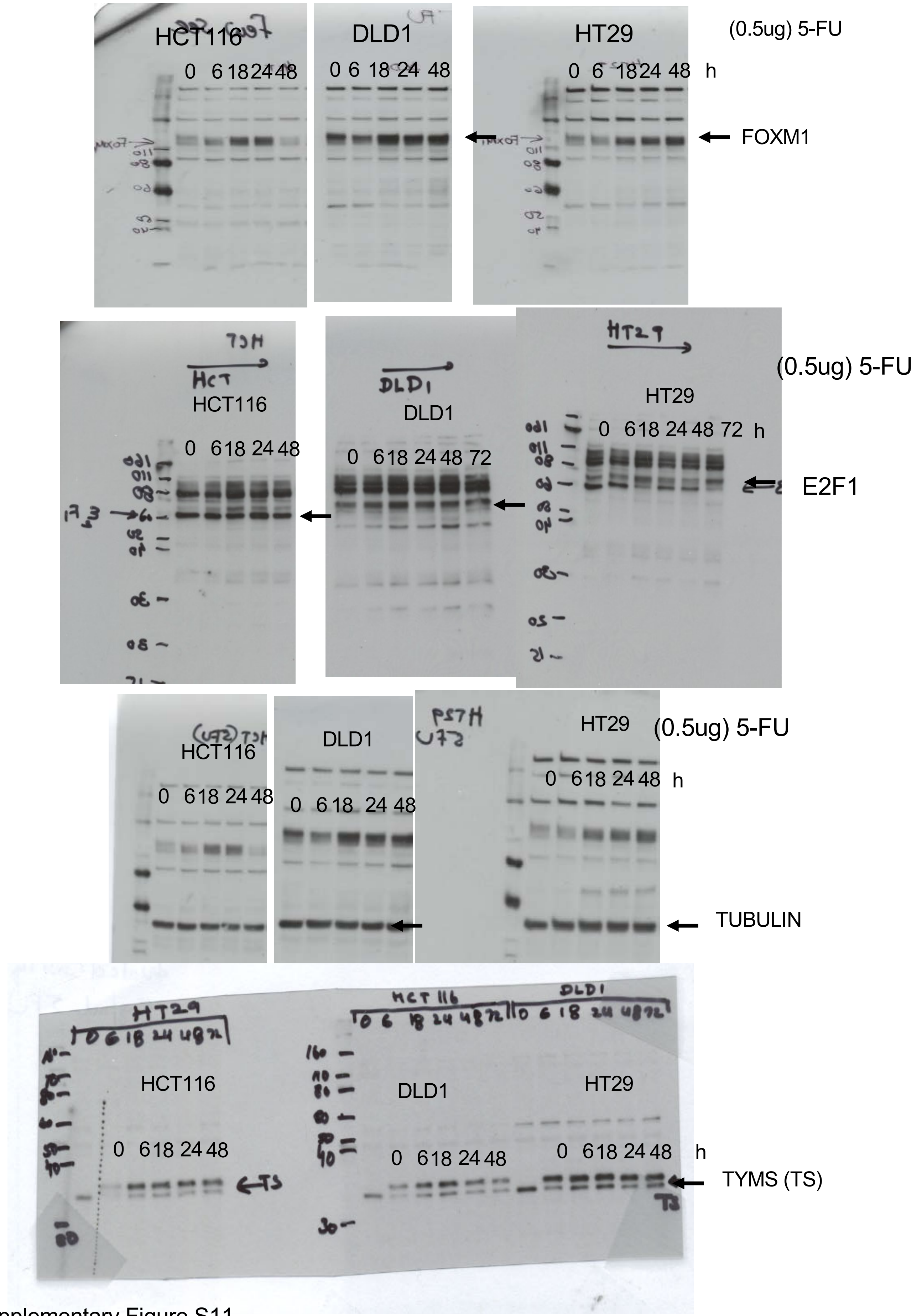

Supplementary Figure S11

Original blots for the cropped/composite Fig 1D

Supplementary Figure S11 (continued)

DLD1

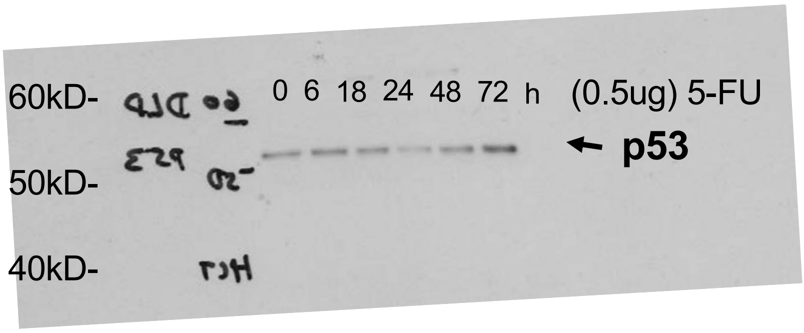

HCT116

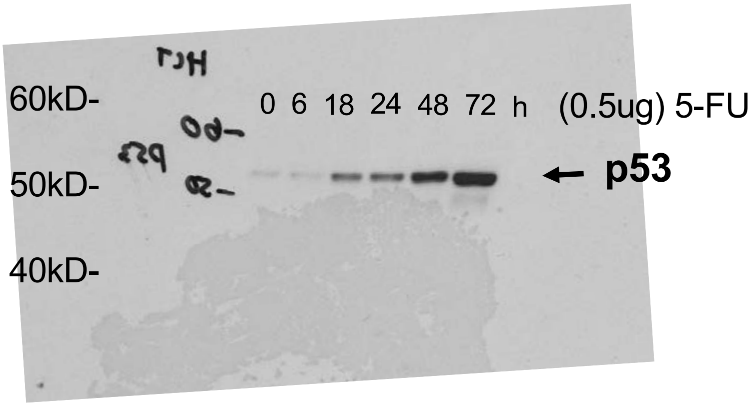

HT29

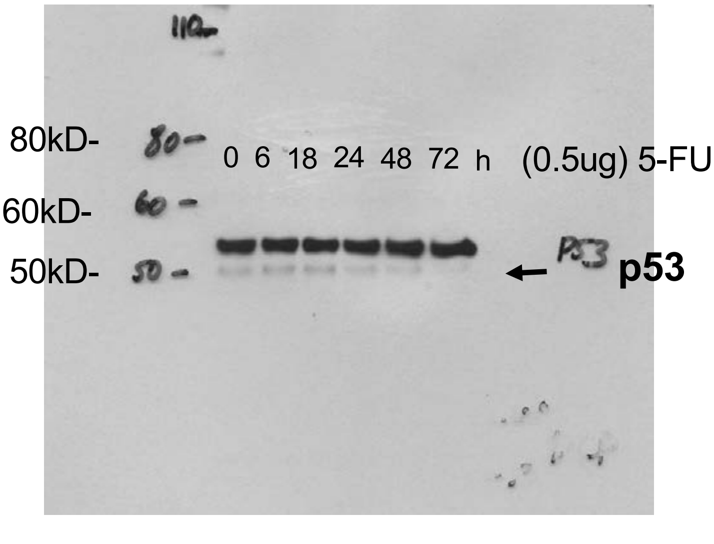

Supplementary Figure S11
Original blots for the cropped/composite Fig 1D

Supplementary Figure S12

HCT116

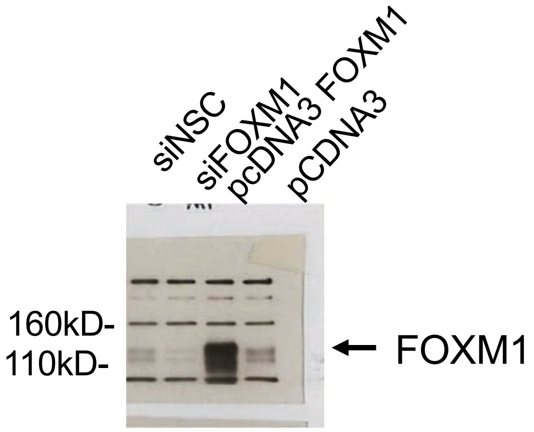

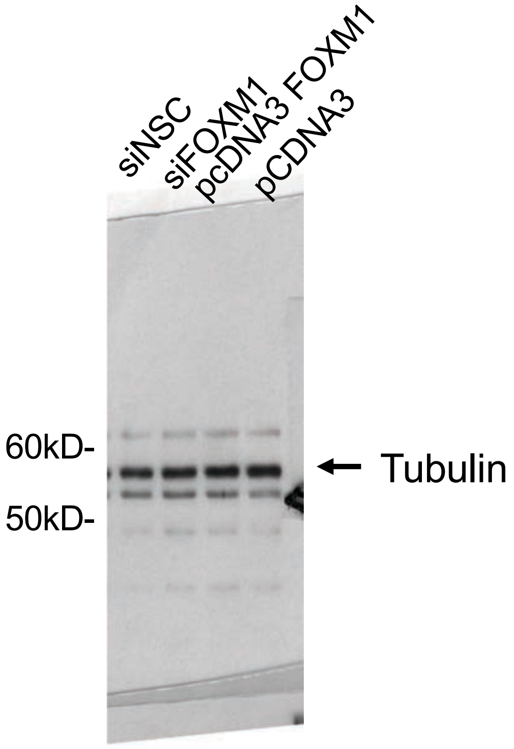

Supplementary Figure S12.
Original blots for the cropped/composite Fig. 2A

Supplementary Figure S12 (continued)

HCT116

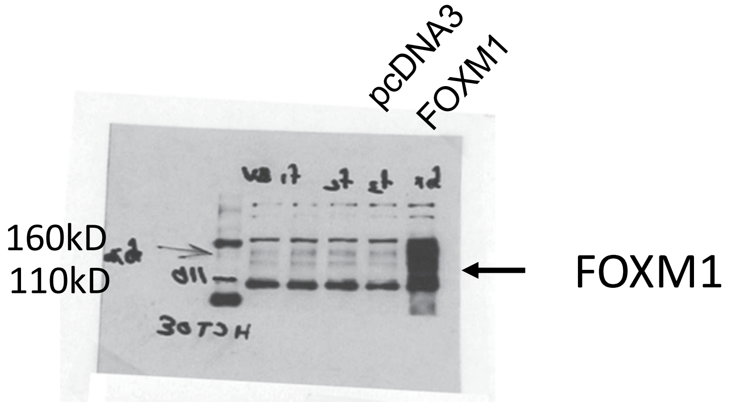

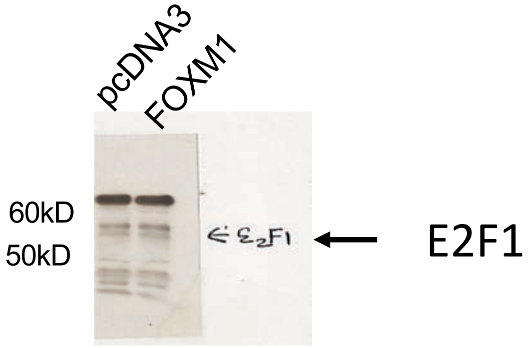

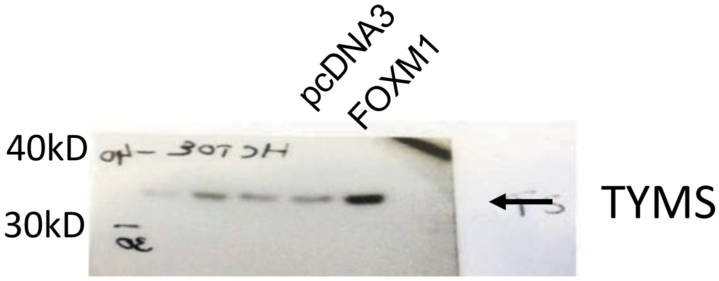

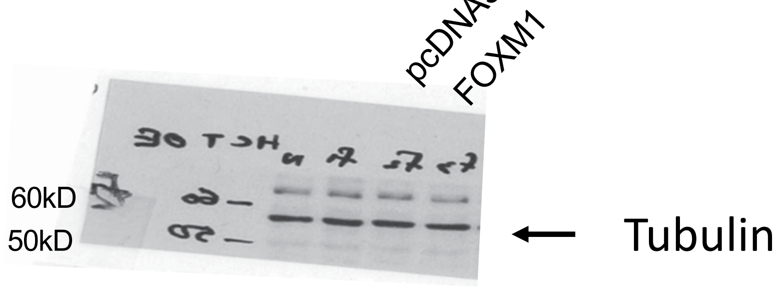

Supplementary Figure S12.

Original full-length blots for the cropped/composite Fig. 2D

Supplementary Figure S12 (continued)

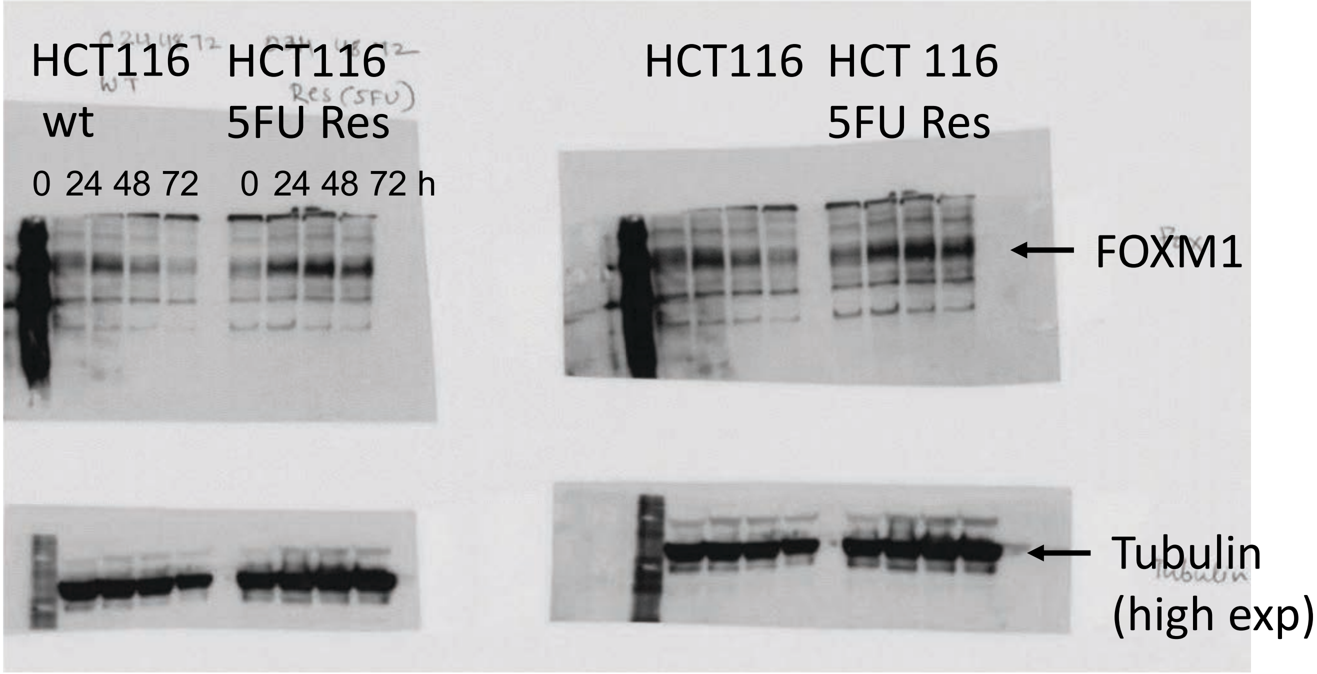

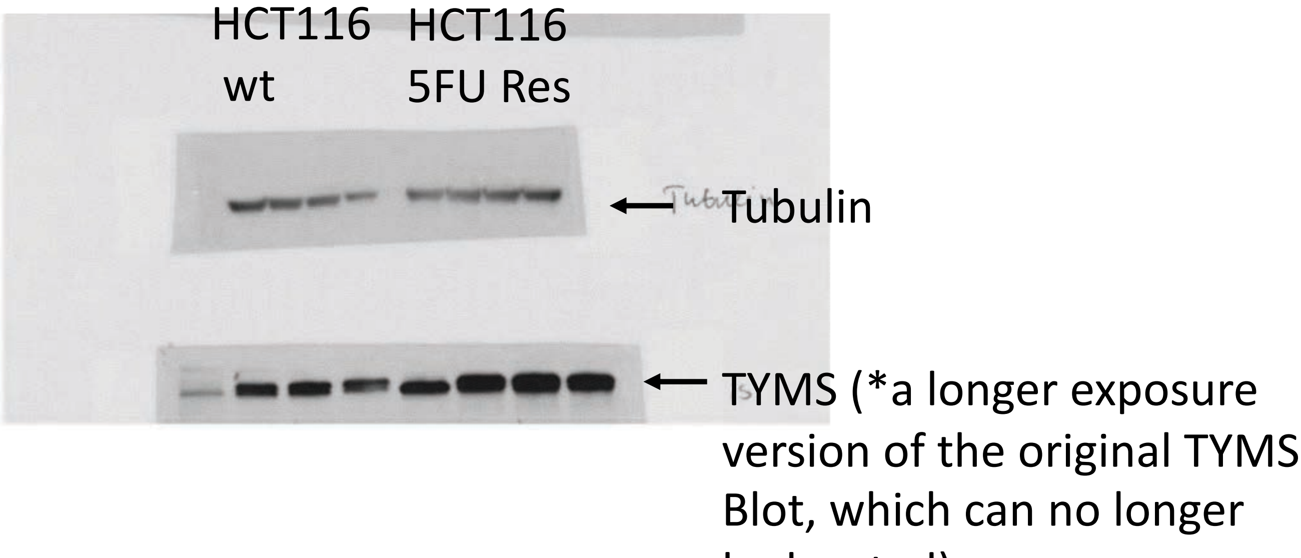

Supplementary Figure S12.
Original full-length blots for the cropped/composite Fig. 2E
* The TYMS blot shown here is a longer exposure version of the original TYMS Blot, which can no longer be located)

Supplementary Figure S13

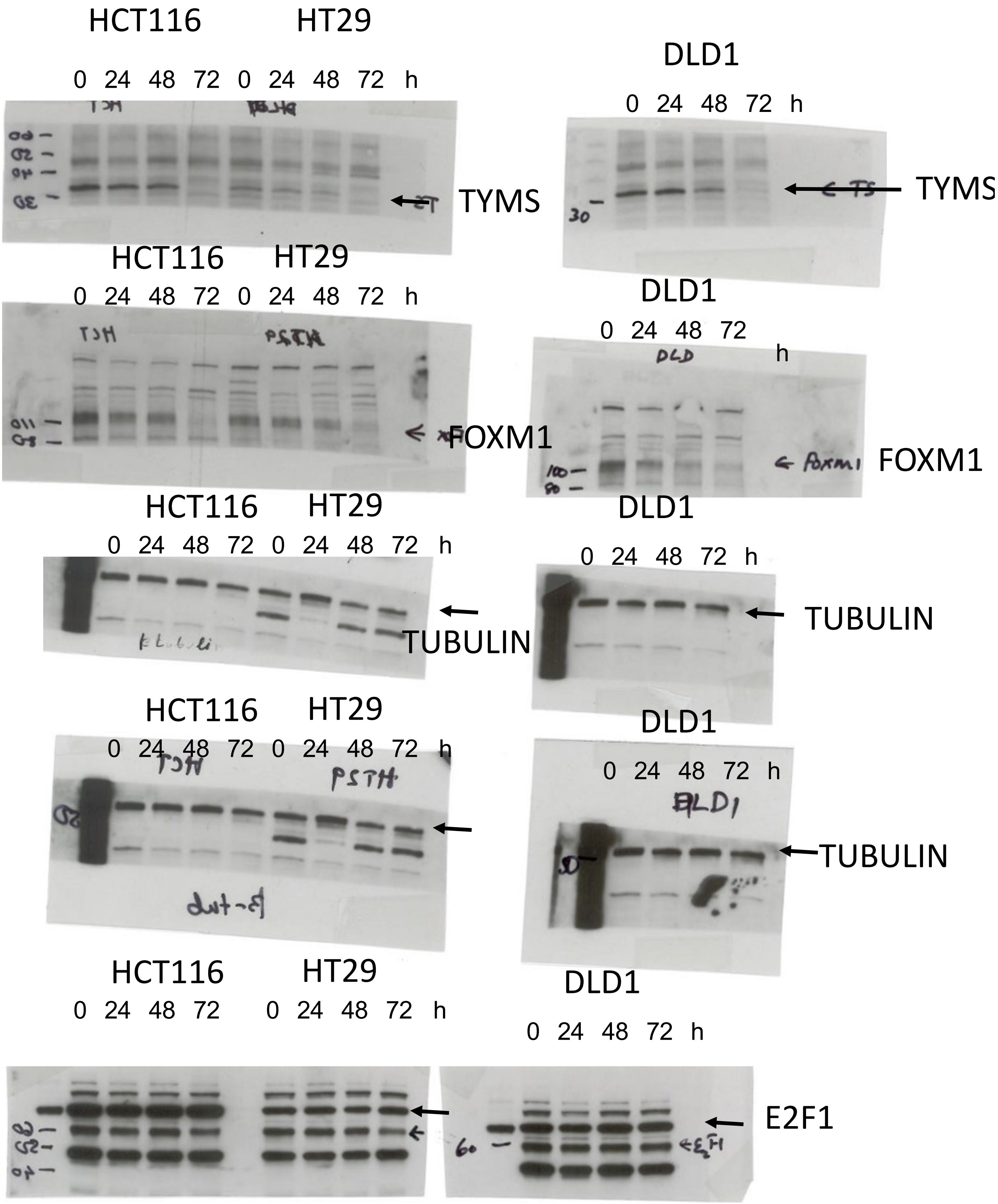

Supplementary Figure S13.

Original full-length blots for the cropped/composite Fig. 3B

Supplementary Figure S14

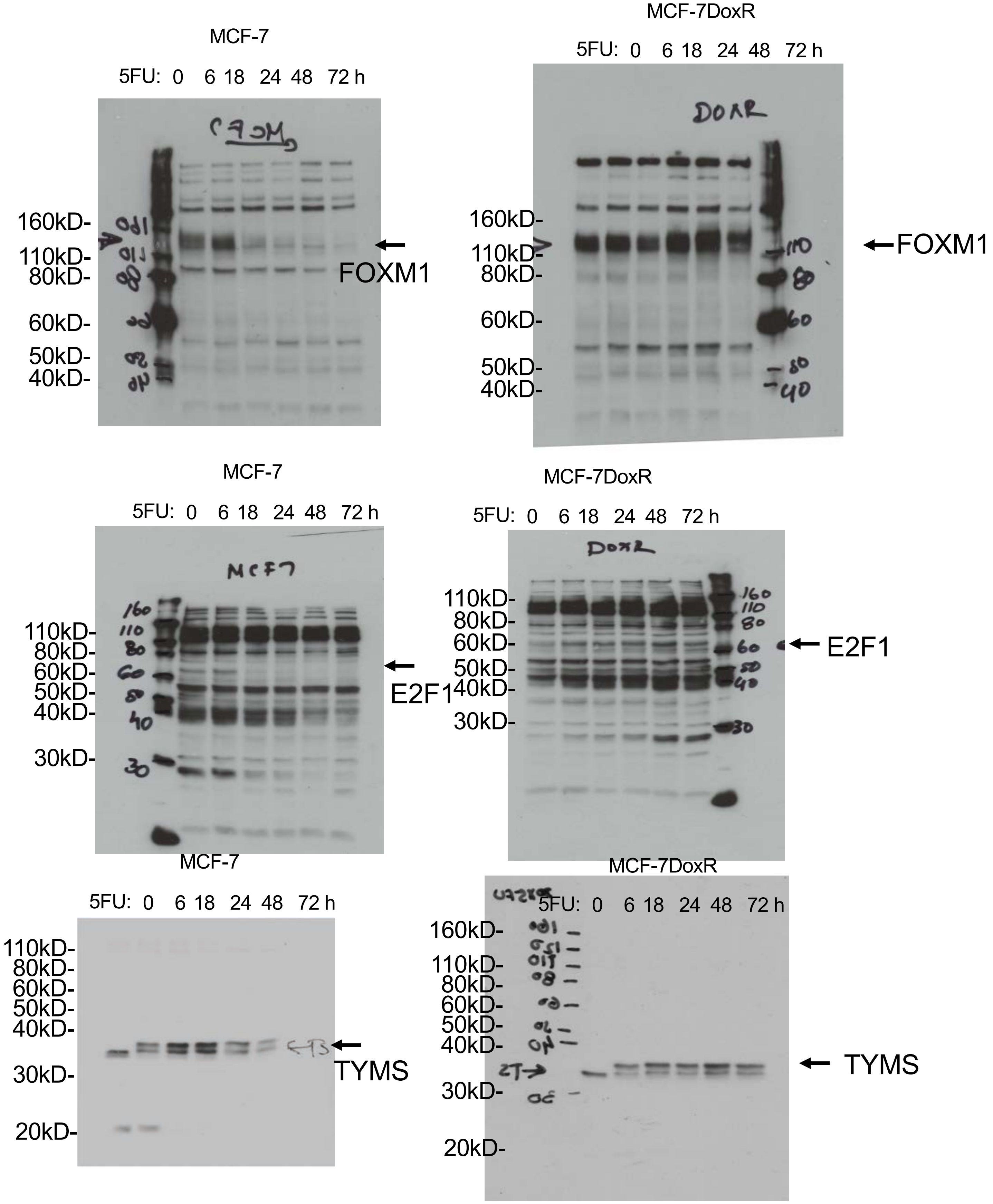

Supplementary Figure S14.

Original full-length blots for the cropped/composite Fig. S1

Supplementary Figure S14

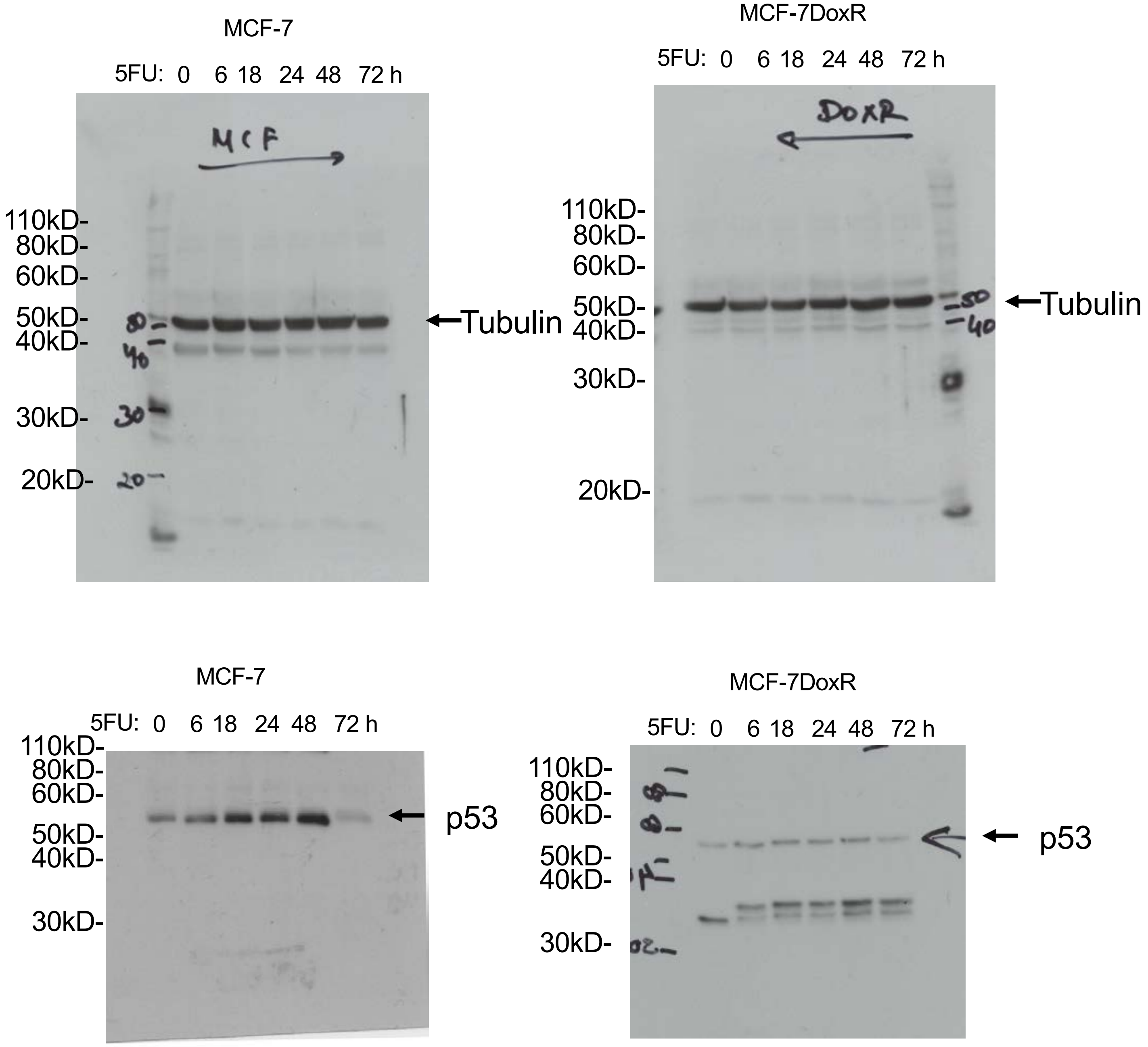

Supplementary Figure S14.

Original full-length blots for the cropped/composite Fig. S1

Supplementary Figure S15

Thiostrepton

HCT118WT HCT118 5-FU Res

0 24 48 72 0 24 48 72 h

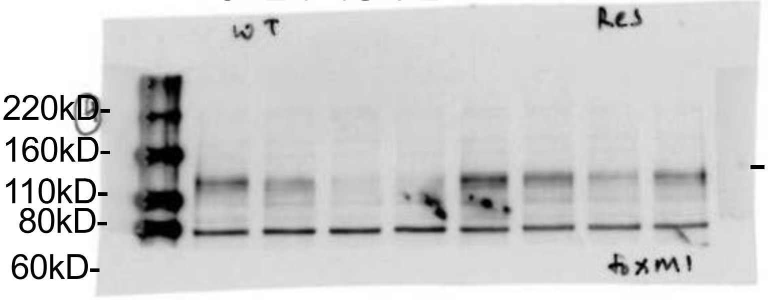

220kD-
160kD-
110kD-
80kD-
60kD-

-FOXM1

Thiostrepton

HCT118WT HCT118 5-FU Res

0 24 48 72 0 24 48 72 h

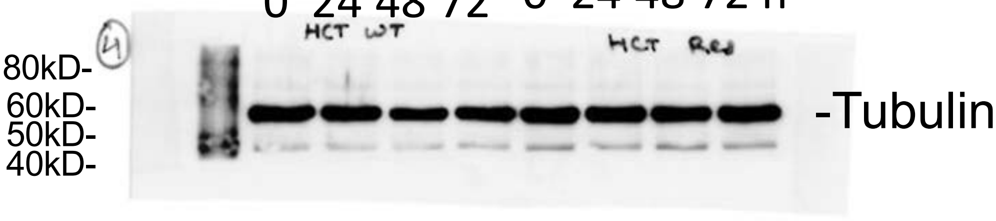

80kD-
60kD-
50kD-
40kD-

-Tubulin

Supplementary Figure S15.

Original full-length blots for the cropped/composite Fig. S2

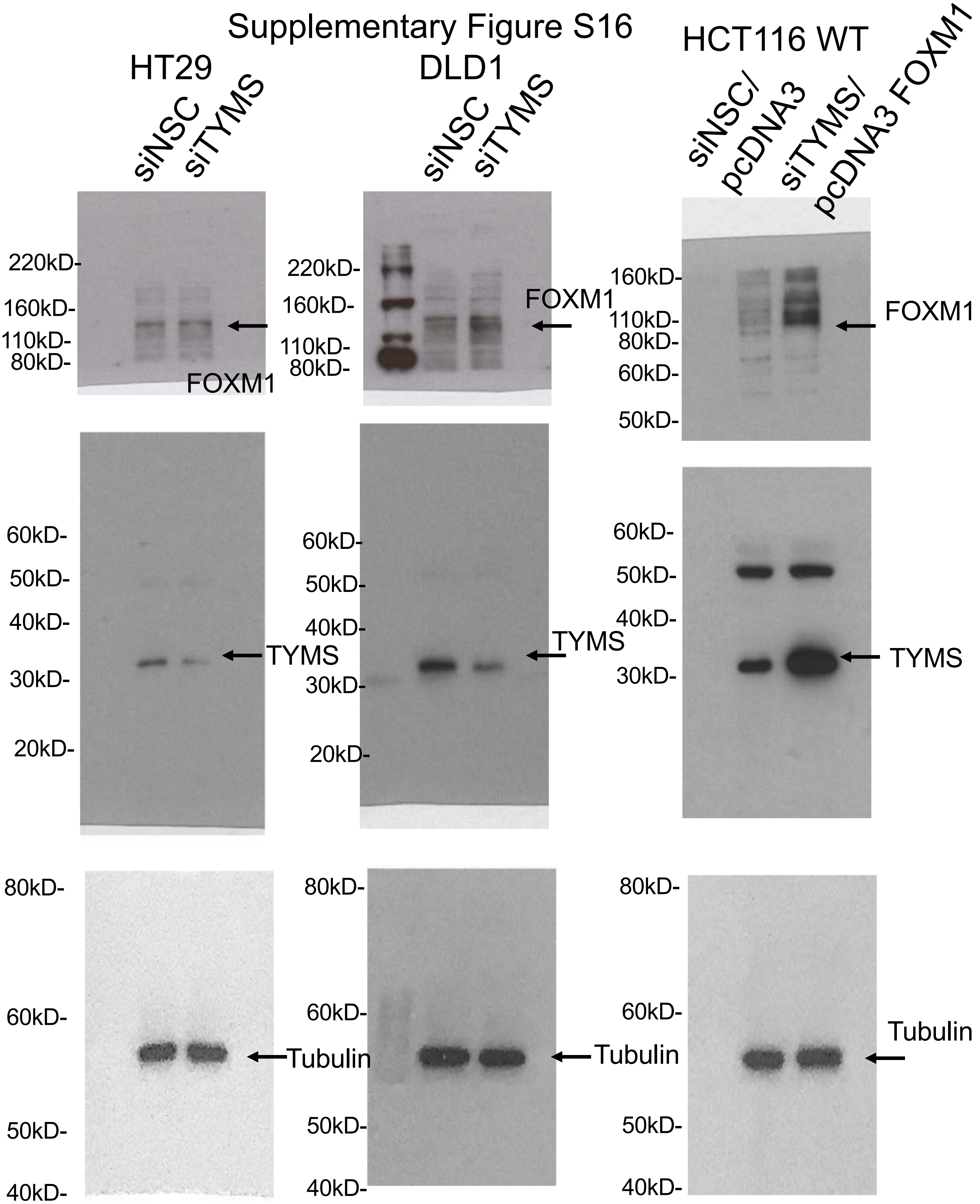

Supplementary Figure S16.

Original full-length blots for the cropped/composite Fig. S3

Supplementary Figure S17

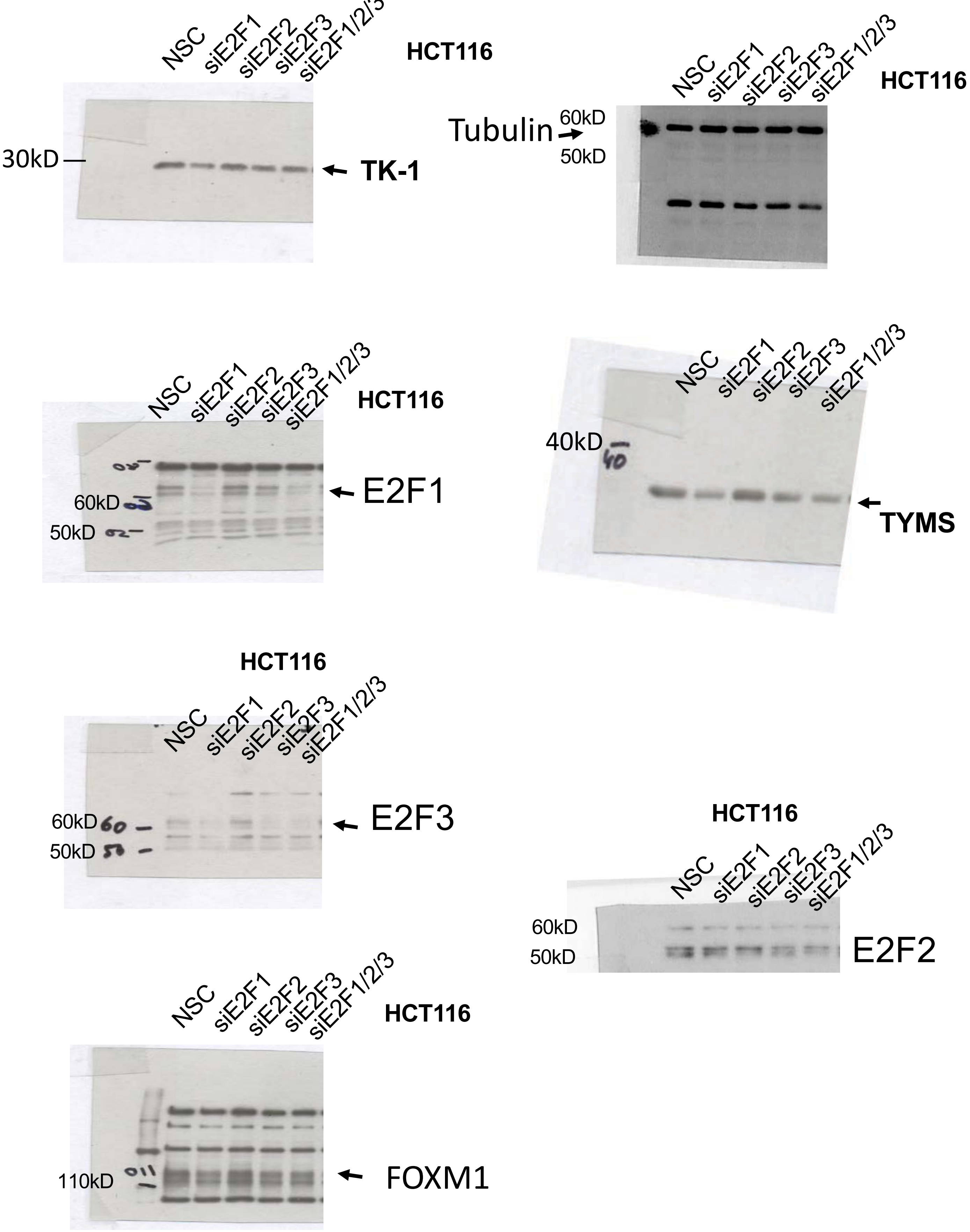

Supplementary Figure S17.

Original full-length blots for the cropped/composite Fig. S9
